# Map-1a regulates Sertoli cell BTB dynamics through the cytoskeletal organization of microtubule and F-actin

**DOI:** 10.1186/s12958-024-01204-y

**Published:** 2024-04-03

**Authors:** Lingling Wang, Ming Yan, Tiao Bu, Xiaolong Wu, Linxi Li, Bruno Silvestrini, Fei Sun, C. Yan Cheng, Hao Chen

**Affiliations:** 1 Institute of Reproductive Medicine, Medical School of Nantong University, Nantong, 226001 Jiangsu China; 2 Jiangsu Key Laboratory of Drug Screening, China Pharmaceutical University, Nanjing, 210009 Jiangsu China; 3 Department of Urology and Andrology, Sir Run Run Shaw Hospital, Zhejiang University School of Medicine, Hangzhou, 310016 Zhejiang China; 4 The Second Affiliated Hospital and Yuying Children’s Hospital, Wenzhou Medical University, Zhejiang 325027, Wenzhou, China; 5 Faculty of Pharmacy, University of Rome La Sapienza, P. Le Aldo Moro 5, 00185 Rome, Italy

**Keywords:** Testis, Spermatogenesis, Sertoli cell, RNAi, Microtubule, Cytoskeleton

## Abstract

Microtubule-associated protein 1a (Map1a) is a microtubule (MT) regulatory protein that binds to the MT protofilaments in mammalian cells to promote MT stabilization. Maps work with MT cleavage proteins and other MT catastrophe-inducing proteins to confer MT dynamics to support changes in the Sertoli cell shape to sustain spermatogenesis. However, no functional studies are found in the literature to probe its role in spermatogenesis. Using an RNAi approach, coupled with the use of toxicant-induced testis (in vivo)- and Sertoli cell (in vitro)-injury models, RNA-Seq analysis, transcriptome profiling, and relevant bioinformatics analysis, immunofluorescence analysis, and pertinent biochemical assays for cytoskeletal organization, we have delineated the functional role of Map1a in Sertoli cells and testes. Map1a was shown to support MT structural organization, and its knockdown (KD) also perturbed the structural organization of actin, vimentin, and septin cytoskeletons as these cytoskeletons are intimately related, working in concert to support spermatogenesis. More importantly, cadmium-induced Sertoli cell injury that perturbed the MT structural organization across the cell cytoplasm was associated with disruptive changes in the distribution of Map1a and a surge in p-p38-MAPK (phosphorylated p38-mitogen-activated protein kinase) expression but not total p38-MAPK. These findings thus support the notion that p-p38-MAPK activation is involved in cadmium-induced Sertoli cell injury. This conclusion was supported by studies using doramapimod, a specific p38-MAPK phosphorylation (activation) inhibitor, which was capable of restoring the cadmium-induced disruptive structural organization of MTs across the Sertoli cell cytoplasm. In summary: this study provides mechanistic insights regarding restoration of toxicant-induced Sertoli cell and testis injury and male infertility.

**Supplementary Information:**

The online version contains supplementary material available at 10.1186/s12958-024-01204-y.

## Introduction

Microtubule-associated proteins (Maps) are comprised of at least eight families of structural and functional proteins that bind onto microtubules (MTs) to modulate MT dynamics through different mechanisms [1–3]. For instance, Map1a, Map1b, Map2, Map4, and Tau bind onto MT protofilaments to maintain MT stability by conferring MT stabilization [3–5]. However, MARKs (Microtubule Affinity Regulating Kinases) composed of MARK 1, 2, 3, and 4 which are all expressed in the testis (all are non-receptor Ser/Thr protein kinases) but mostly MARK 2 and 4 [6] are capable of phosphorylating Maps (e.g., Map2, Map4, and Tau), causing their detachment from microtubules that induce MT catastrophe [4, 7, 8]. As such, MAPs and MARKs are working in concert to maintain MT dynamics to support the cytoskeletal organization of MTs in mammalian cells. Besides the notable function of Maps mentioned above, Map families of proteins are also involved in modulating MT dynamics [3, 4, 8, 9]. First, Maps promote MT stability by blocking MTs from being cleaved by katanins and spastins, leading to MT disruption and catastrophe. Second, Maps (e.g., Map1S and Map1a) induce cytoskeletal cross-linking of protofilaments of MTs and actin filaments to promote cytoskeletal stability. Third, Maps (e.g., Tau, Map1b) promote MT anchorage onto the plasma membrane. Fourth, Maps induce either parallel or anti-parallel bundling (e.g., Map65) of polarized microtubules. Fifth, Maps (e.g., Map4, Tau) maintain proper MT spacing. Furthermore, structural Maps that bind onto protofilaments of MTs also modulate the function of MT-dependent motor proteins (e.g., dynein 1, kinesins) by either activating motor protein function or serving as roadblocks to inactivate motor proteins (e.g., Map7) [3, 10–13]. Importantly, while MTs in Sertoli cells are crucial to support spermatogenesis across the seminiferous epithelium of the mammalian testis [8, 14–16], few studies investigated the functional and mechanistic roles of Maps on spermatogenesis in the testis. We attempted to fill this knowledge gap by studying proteins that are crucial to support MT dynamics such as the MT-dependent motor proteins dynein 1 [17] and kinesin 15 [18], as well as MARK 4 [6].

Based on our initial preliminary studies investigating the role of MTs in testis function using the adjudin model, Map1a and Map1b are expressed by Sertoli cells and they are the two predominant structural Maps in the testis [19, 20]. Other studies using genetic models have shown that deletion of Map1a in *Map1a*^−/−^ mice led to learning and memory disturbances as a result of defects in neuron synaptic function [21]. Furthermore, spontaneous mutation of nm2719 that disrupts the *Map1a* gene in mice displayed the phenotypes of tremors and ataxia, affecting coordination, balance, and speech due to the loss of cerebellar Purkinje neurons in these adult mice [22]. However, any defects in male fertility were not reported since the testis was not examined in these earlier studies. On the other hand, Map1a protein-truncating variants were found to be associated with autism spectrum disorder and attention deficit hyperactivity disorder in humans [23], supporting the notion that this protein is crucial to support the neuronal cytoskeletal organization of microtubule. Yet its function in the testis remains unexplored. In this report, our aim is to investigate the role of Map1a in Sertoli cell function through its effects on MT and other cytoskeletons, and the mechanistic basis of its regulatory role.

## Materials and methods

### Animals and ethics statement

Male Sprague–Dawley rats at 18 days of age in groups of 10 were obtained with a foster mother. Adult male Sprague–Dawley rats at ~ 250 g body weight (b.w.) were used. All rats were purchased from The Laboratory Animal Center of Nantong University (Nantong, Jiangsu, China). Rats were housed at the Laboratory Animal Center of Nantong University at 20 ± 1 °C with a 12 h of light and 12 h of dark cycle. All rats had free access to water and standard rat chow. Pups were used at 20 days of age for the isolation of Sertoli cells for primary cultures after at least 48 hr of rest. Adult male Sprague–Dawley rats were used for studies involving the cadmium model, and for morphological analysis, including histological analysis and immunohistochemistry (IHC), to examine changes in the distribution of Map1a across the seminiferous epithelium during the epithelial cycle as described [20]. Adult male rats were also used for the isolation of total germ cells for some experiments using protocols as detailed elsewhere [24]. All experiments were approved by the Laboratory Animal Ethics Committee of Nantong University with Protocol Numbers S20221015-1001, S20240116-006, and S20240116-007. Rats were euthanized by CO_2_ asphyxiation using a slow (20%-30%/min) displacement of chamber air from a compressed CO_2_ tank with a gas regulator that regulated the airflow into a euthanasia chamber which was built and approved by Laboratory Animal Center of Nantong University.

### Antibodies, primer sequences, and siRNAs for RNAi

Antibodies used for our experiments were obtained commercially except otherwise specified (Table 1 and Table S1). The RRID (Resource Identification Initiative) numbers, vendors and working dilutions of all antibodies are found in Table 1 and Table S1. Antibodies noted in Table S1 were used for IB analysis reported in Figures S1 and S2. The nucleotide sequences of primer pairs for RT-PCR (Real-time PCR)/qPCR (quantitative real-time PCR) and siRNA duplexes used for RNAi are listed in Tables 2 and 3, respectively.

**Table 1:** Antibodies used for different experiments in this report

Antibody (RRID)	Host Species	Vendor	Catalog Number	Working dilution	
				**IB Analysis**	**IF/IHC Analysis**
Map1a (AB_2732015)	Rabbit	Abcam	ab184350	1:250	—
Map1a (AB_10903974)	Rabbit	Abcam	ab101224	1:125	1:125 (cells) 1:50 (IHC)
Map1a	Rabbit	Abcam	ab184349	—	1:50 (tissue)
Map2 (AB_2137880)	Rabbit	Proteintech	17490–1-AP	1:500	—
Occludin (AB_2533977)	Rabbit	Thermo Fisher Scientific	71–1500	1:300	—
JAM-A (AB_2533241)	Rabbit	Thermo Fisher Scientific	36–1700	1:500	1:100
ZO-1 (AB_10733242)	Rabbit	Proteintech	21773–1-AP	1:500	—
ZO-1 (AB_2533147)	Mouse	Thermo Fisher Scientific	33–9100	—	1:100
N-Cadherin (AB_444317)	Rabbit	Abcam	ab18203	1:1000	—
N-Cadherin (AB_2313779)	Mouse	Thermo Fisher Scientific	33–3900		1:100
β-Catenin (AB_2533982)	Rabbit	Thermo Fisher Scientific	71–2700	1:500	1:100
Detyrosinated-α-Tubulin (AB_869990)	Rabbit	Abcam	ab48389	1:500	1:100
Acetylated-α-Tubulin (AB_448182)	Rabbit	Abcam	ab24610	1:500	1:100
Tyrosinated-α-Tubulin (AB_261811)	Mouse	Sigma-Aldrich	T9028	1:500	1:100
Dynein1 (Heavy chain, Dync1h1)	Mouse	Santa Cruz	sc-514579	1:250	—
Dynein1 (Heavy chain, Dync1h1) (AB_2261765)	Rabbit	Proteintech	12345–1-AP	—	1:100
Kif15 (AB_11182836)	Rabbit	Proteintech	55407–1-AP	1:500	1:100
EB1 (AB_397891)	Mouse	BD Biosciences	610534	1:250	1:100
CAMSAP2 (AB_2068826)	Rabbit	Proteintech	17880–1-AP	1:500	1:100
α-Tubulin (AB_2241126)	Rabbit	Abcam	Ab7291	1:500	1:100
β-Tubulin (AB_2210370)	Rabbit	Abcam	Ab6046	1:500	1:100
Arp3 (AB_476749)	Mouse	Sigma-Aldrich	A5979	1:500	1:100
Eps8 (AB_397544)	Mouse	BD Biosciences	610143	1:500	1:100
Palladin (AB_2158782)	Rabbit	Proteintech	10853–1-AP	1:250	—
Annexin A2 (AB_11129437)	Rabbit	Cell Signaling Technology	8235	1:500	—
Vimentin (AB_628437)	Mouse	Santa Cruz	sc-6260	1:500	1:100
Septin7 (AB_2254298)	Rabbit	Proteintech	10818–1-AP	1:250	1:100
GAPDH (AB_2107436)	Mouse	Proteintech	60004–1-Ig	1:3000	—
β-actin (AB_630836)	Mouse	Santa Cruz Biotechnology	sc-4778	1:1000	—
Goat Anti-Mouse IgG H&L (Alexa Fluor® 680)	Mouse	Abcam	ab175775	1:3000	—
Goat Anti-Rabbit IgG H&L (IRDye® 800)	Rabbit	Abcam	ab216773	1:3000	—
Goat Anti-Rabbit IgG H&L (Alexa Fluor® 680)	Rabbit	Abcam	ab175773	1:3000	—
Goat Anti-Mouse IgG H&L (IRDye® 800)	Mouse	Abcam	ab216772	1:3000	—
Rhodamine Phalloidin (Molecular Probes™)		Thermo Fisher Scientific	R415	—	1:200
Mouse IgG Alexa Fluor 488 (AB_2534088)	Goat	Thermo Fisher Scientific	A-11029	—	1:250
Mouse IgG Alexa Fluor 555 (AB_141780)	Goat	Thermo Fisher Scientific	A-21424	—	1:250
Rabbit IgG Alexa Fluor 488 (AB_2576217)	Goat	Thermo Fisher Scientific	A-11034	—	1:250
Rabbit IgG Alexa Fluor 555 (AB_2535850)	Goat	Thermo Fisher Scientific	A-21429	—	1:250

**Table 2:** Primer pairs used for RT-PCR and qPCR

Gene name	Accession number	Orientation	Nucleotide sequence	Nucleotide position	Product Length (bp)	Annealing Temp (℃)
Map1a	NM_030995.2	Sense Anti-Sense	5’-AAACGGAGGCTGATCAAGGA-3’ 5’-TTCAGGTCTGGCTTGGAGAG-3’	1174–1193 1375–1394	221	60.0
Map1b	NM_019217.1	Sense Anti-Sense	5’-GGAAGAAGACGGGGAAGACA-3’ 5’-GTCCTCTGCTTTGTCCTCCT-3’	2841–2860 3047–3066	226	60.0
Map1s	NM_001106070.1	Sense Anti-Sense	5’-CCCTGGAGAAGCAGAGAACA-3’ 5’-GACTCGTCCACCTCAGTTGA-3’	1653–1672 1873–1892	240	60.0
Map2	NM_013066.1	Sense Anti-Sense	5’-TCTCAGTTCAGTGCCAGAGG-3’ 5’-TTTCCTGAGGTGCTTCGGAT-3’	3051–3070 3252–3271	221	60.0
Map4	NM_039082634.1	Sense Anti-Sense	5’-GGGTGTGTTTTTGAGCAGGT-3’ 5’-GCAGGCTCCAAGCATAGTTC-3’	8387–8406 8566–8585	199	60.0
Map6	NM_017204.1	Sense Anti-Sense	5’-CAGCTACAGTGCCCAGTTCA-3’ 5’-TCTGGCTCGTTGAGGTCTTT-3’	2026–2045 2179–2198	173	60.0
Map7	NM_001106270.2	Sense Anti-Sense	5’-GAATGGAATTTCAGGCTCCA-3’ 5’-ATGCCACCTCAGGGTATCAG-3’	2468–2487 2629–2648	181	60.0
Map9	NM_001135716.1	Sense Anti-Sense	5’-TGATCAAGACGTTGGCAGAG-3’ 5’-GGGCAGAATCTTCGGTTGTA-3’	608–627 812–831	224	60.0
Map10	NM_001277391.1	Sense Anti-Sense	5’-ATTTTGCCCTCCTCCTCTGT-3’ 5’-CCCTGCATCTGTGGAAGATT-3’	805–824 1007–1026	222	60.0
Map6d1	NM_001108844.1	Sense Anti-Sense	5’-GAAGCCCTCAAGATCCACAA-3’ 5’-GAGGGGCTGATGTCTGAAAG-3’	416–435 548–567	152	60.0
Map7d3	NM_001271327.1	Sense Anti-Sense	5’-TCCTGTTGTCAGCTTGGATG-3’ 5’-GGTGCTTGTTTCTTGGTGGT-3’	1107–1126 1336–1355	249	60.0
Kif15	NM_181635.2	Sense Anti-Sense	5’-CAGCTTCAGCTGGACAATGC-3’ 5’-TGTTGCGCTCTTTTTCTGCC-3’	2547–2566 2741–2760	214	60.0
S16	NM_001169146.1	Sense Anti-Sense	5’-TCCGCTGCAGTCCGTTCAAGTCTT-3’ 5’-GCCAAACTTCTTGGATTCGCAGCG-3’	87–110 448–471	385	60.0

**Table 3:** siRNA duplexes used for RNAi experiments

Gene	Product	Catalog no	Target sequence (5’ – 3’)
Map1a	Map1a siRNA duplexes	AM16704	Sense: GCCUUGCUGCUACAUCUUUtt
			Antisense: AAAGAUGUAGCAGCAAGGCtt
Non-Target	ON-TARGETplus Non-targeting Pool siRNA duplexes	D-001810–10	UGGUUUACAUGUCGACUAA
			UGGUUUACAUGUUGUGUGA
			UGGUUUACAUGUUUUCUGA
			UGGUUUACAUGUUUUCCUA

### Isolation of Sertoli cells and primary Sertoli cell cultures

Sertoli cells were isolated from the testes of 20-day-old male pups for primary cultures as earlier described [25] and detailed elsewhere in our laboratory [26]. Freshly isolated Sertoli cells were seeded on Matrigel (BD Biosciences, San Jose, CA)-coated culture dishes (either 6-, 12-, or 24-well dishes), coverslips (to be placed in 12-well dishes), and bicameral units (Millipore, Burlington, MA; to be placed in 24-well dishes). Cells were incubated at a density of 0.3–0.4 (for IB (immunoblotting)), 0.03–0.04 (for IF (immunofluorescence analysis)), and 1.0–1.2 (for TER (transepithelial electrical resistance measurement)) × 10^6^ cells/cm^2^, respectively, in serum-free DMEM (Dulbecco’s Modified Eagle Medium)/F-12 (Ham’s F12 Nutrient Mixture) (Sigma-Aldrich, St. Louis, MO) medium. DMEM/F12 medium was supplemented with bovine insulin (10 µg/mL), human transferrin (5 µg/mL), EGF (epidermal growth factor, 2.5 ng/mL), bacitracin (5 µg/mL), and gentamicin (20 µg/mL). These cell densities were selected based on pilot experiments based on the following rationale. First, sufficient proteins in lysates of Sertoli cells were used for IB or biochemical assays that monitored changes in cytoskeletal organization of microtubules or F-actin using cells cultured on 6- and 12-well dishes, containing 5- and 3-ml DMEM/F12 medium, respectively. Second, changes in protein distribution at the Sertoli cell–cell interface or across the cell cytoplasm (such as distribution of actin filaments or MT protofilaments) were notably detected by fluorescent microscopy with coverslips placed in 12-well dishes, and each well contained 2-ml DMEM/F12 medium. Third, the Sertoli cell tight junction (TJ)-barrier was readily detected when cells were cultured on the bicameral units, which were placed in 24-well dishes, with 0.5-ml DMEM/F12 medium in the apical and basal compartment. The assembly of TJ was monitored by sending a current across the cell epithelium which was capable of blocking the current by measuring the conductivity in Ohms. Sertoli cells were incubated in a humidified CO_2_ incubator with 95% air/5% CO_2_ (vol/vol) at 35 °C. Depending on the experiments as noted in the treatment regimens, Sertoli cells were used on day 3 after a functional TJ-barrier was established including the presence of ultrastructures of TJ, basal ES, and gap junction [27–29].

### Treatment of rats or Sertoli cells with CdCl_2_

CdCl_2_ from Sigma-Aldrich dissolved in saline (0.9% NaCl in MilliQ water) at 1 mM or 3 mM served as a stock solution. Sertoli cells cultured for 3 days were treated with 0.9% NaCl (control) *versus* CdCl_2_ at 1 µM for 6 h for IF or at 3 µM for 24 h for IB, qPCR, and RNA-Seq for transcriptome profiling. The selection of these concentrations and treatment times for specific experiments reported herein was based on the results of a series of pilot experiments, and published in earlier reports [25, 30–32], to obtain the notable phenotypes without impeding cell viability. For in vivo experiments, adult rats were treated with CdCl_2_ at 3 mg/kg b.w. via intraperitoneal injection (i.p.) using a stock solution of 10 mg/ml as described [33]. Rats in groups of 4 to 6 rats were sacrificed by CO_2_ asphyxiation at 0, 6, 12, 24, 48, 72, and 96 h thereafter. As such, at least *n* = 3 testes from different rats were used for different experiments. Testes were snap-frozen in liquid nitrogen and stored at -80℃ until use. In experiments for histological analysis (by hematoxylin and eosin staining) or IF analysis, testes were fixed in modified Davidson’s fixative, embedded in paraffin to obtain 5-μm thick sections using a microtome as described [34, 35].

### Map1a Knockdown (KD) by RNA interference (RNAi) in Sertoli Cells Cultured In Vitro

Map1a KD was performed by RNAi using Sertoli cells cultured on day 3 when a functional TJ-permeability barrier that mimicked the blood-testis barrier (BTB) in vivo was established. Sertoli cells were transfected with specific Map1a small interfering RNA (siRNA) duplexes vs non-targeting negative control (Ctrl RNAi) siRNA duplexes (Table 3) on day 3 at 100 nM (for IB, IF, and polymerization/spin-down assay) for 24 h using RNAiMAX (Life Technologies, Carlsbad, CA) as a transfection reagent. For the assessment of Sertoli TJ-barrier function, TER across the Sertoli cell epithelium was measured daily, and transfection was performed on Day 3 when a steady TER was achieved. Map1a siRNA duplexes were obtained from Ambion (Ambion Corp, Naugatuck, CT, USA). Transfection medium and reagents were removed from Sertoli cells by rinsing cultures thrice with DMEM/F12. Using the regimen reported here, it was able to obtain ~ 70% Map1a KD routinely when the expression was assessed by IB, IF, and/or qPCR. The Sertoli cells were then used for corresponding experiments including qPCR, IF, IB, BTB integrity assay or actin, and MT polymerization assays. For Sertoli cell cultures to be used for IF, cells were co-transfected with 1 nM siGLO red transfection indicator (Dharmacon) to track successful transfection. In each experiment, replicates or triplicates of cultures were used for each treatment and control group. Data reported here were representative results from *n* = 3 independent experiments using different batches of Sertoli cells and yielded similar results. All samples within an experiment, including both treatment and control groups, were analyzed simultaneously to avoid inter-experimental variations

### Assessment of Sertoli cell TJ-permeability barrier by quantifying transepithelial electrical resistance (TER) across the Sertoli cell epithelium in vitro

Sertoli cells cultured in vitro assembled an intact epithelium with a functional TJ-barrier, capable of resisting the conductivity of short electrical current delivered by a Millipore Millicell-ERS (electrical resistance system). This resistance was then recorded by quantifying the TER) in ohms (Ω) by placing two electrodes across the Sertoli cell epithelium, one in the apical and the other in the basal compartment of the bicameral units. In brief, Sertoli cells were plated on Matrigel-coated bicameral units (diameter 12 mm; pore size 0.45 µm, effective surface area 0.6 cm^2^; EMD Millipore) at 1.0 ~ 1.2 × 10^6^ cells/cm^2^. Each bicameral unit was placed inside a single well of the 24-well dish with 0.5-mL F12/DMEM each in the apical and the basal compartments. Transfections of Map1a *versus* non-targeting control siRNA duplexes **(**Table 3**)** were performed on day 3 at 100 nM for 24 h. Sertoli cell TJ-permeability was recorded daily before and after transfection until the end of the experiment. In each of the *n* = 3–4 experiments, each treatment *versus* control groups had quadruple bicameral units.

### BTB integrity assay in vitro

Sertoli cell BTB integrity in vitro was further assessed by the ability of a functional BTB to block the passage of a small membrane-impermeable biotin, EZ-LinkSulfo-NHS-LC-Biotin (Mr 556.59; Thermo Fisher Scientific, Waltham, MA) as described [25, 36] and detailed elsewhere [37]. In brief, freshly isolated Sertoli cells were plated on Matrigel-coated round coverslips (placed in 12-well dishes) at a density of 0.03 ~ 0.04 × 10^6^ cells/cm^2^ so the cell–cell interface in the epithelium can be distinctively visible by fluorescence microscopy. On day 3, Sertoli cells were transfected with Map1a siRNA duplexes *vs.* non-targeting negative control siRNA duplexes at 100 nM for 24 h **(**Table 3**)**. Thereafter, cells were rinsed thrice on day 4 to remove transfection reagents, and cells were incubated with EZ-Link Sulfo-NHS-LC-Biotin, freshly diluted in 500 μL phosphate-buffered saline (PBS; 10 mM sodium phosphate, 0.15 M NaCl, pH 8.0 at 22 °C) at 0.1 mg/mL containing 0.1 mM CaCl_2_ for 30 min to allow biotinylation. Sertoli cells were then quenched, and the remaining biotin reagent was removed. Biotin that was retained at the BTB or that had penetrated the barrier and found inside the cytoplasm was monitored by Alexa Fluor 488-streptavidin (Life Technologies, 1:250) and co-stained with 4’,6-diamidino-2-phenylindole (DAPI; Sigma) to visualize cell nuclei [25].

### Isolation of germ cells

Total germ cells were isolated from adult male rats (250–300 g body weight) using a nonenzymatic procedure from our laboratory as detailed elsewhere [24]. However, the glass wool filtration step was omitted to retain elongating/elongated spermatids and spermatozoa in the cell preparation [24]. Freshly isolated germ cells, designated total germ cells, were used within 6 h, which had a viability of > 98% when assessed by erythrosine red dye exclusion assay [38], for lysate preparation or total RNA isolation for RT-PCR or qPCR.

### RNA extraction and RT-PCR or qPCR

Total RNA was isolated from rat testes, Sertoli cells, germ cells, and the brain using Trizol™ reagent (Life Technologies) [18]. In brief, 1 µg total RNA was reverse transcribed with PrimeScript™ (Otsu, Japan) 1st Strand cDNA Synthesis Kit according to the manufacturer’s instructions to obtain complementary DNAs (cDNAs). RT-PCR was performed using primer pairs specific to different genes including multiple Maps with S16 serving as the PCR loading control **(**Table 2**)**. The authenticity of PCR products was verified by direct nucleotide sequencing at AZENTA (Suzhou, China). qPCR used to quantify the steady-state mRNA levels in different Maps was performed as described [25, 39] using a LightCycler® 96 real-time PCR system (Roche, Basel, Switzerland). S16 was used as an internal control for normalization, and the specificity of the fluorescence signal was verified by melting curve analysis. The expression level of these genes **(**Table 2**)** gene was determined using the 2^−ΔΔC^_T_ method as described [39].

### RNA sequencing and bioinformatics analysis

The quality of RNA extracted from primary cultures of Sertoli cells including CdCl_2_-treated (24 h) and control cells using Trizol reagent was assessed on an Agilent 2100 Bioanalyzer (Agilent Technologies, Palo Alto, CA, USA) and RNase-free agarose gel electrophoresis. Sertoli cells mRNA enriched by Oligo(dT) beads was fragmented into short fragments using fragmentation buffer and reversely transcribed into cDNAs by using NEBNext Ultra RNA Library Prep Kit for Illumina (NEB 7530, New England Biolabs, Ipswich, MA, USA). The purified double-stranded cDNA fragments were end-repaired, A base was added and ligated to Illumina sequencing adapters. The ligation reaction was purified with the AMPure XP Beads (1.0X). Ligated fragments were subjected to size selection by agarose gel electrophoresis and PCR amplified. The resulting cDNA library was sequenced using Illumina Novaseq6000 in Gene Denovo Biotechnology Co. (Guangzhou, China). Bioinformatics analysis was performed as described [25].

### Immunohistochemistry (IHC), histological analysis (HE), immunofluorescence analysis (IF), cytoskeletal staining of F-actin and microtubules (MTs), and fluorescence image analysis

These analyses were performed and detailed in earlier reports [20, 25, 35, 36, 40]. Images were examined and acquired using a ZEISS Axiocam 503 fluorescence microscope and all image files were saved in TIFF format. Image overlays were performed using Adobe Photoshop CS6 to obtain merge images. Fluorescence image intensity was analyzed using ImageJ 1.45 (National Institutes of Health, Bethesda, MD, USA) software package. All samples from the control and treatment groups were processed and images were acquired in a single experimental session to avoid inter-experimental variations. Data reported in various figures are representative findings of an experiment from a total of at least *n* = 3 independent experiments that yielded similar results, excluding pilot experiments to establish the experimental conditions. For fluorescence intensity or distribution analysis in Sertoli cells or seminiferous tubules of testes, at least 100 cells (or 50 cell-pairs) were randomly selected and examined in experimental and control *versus* experimental groups in a single experimental session to avoid inter-experimental variations.

### Lysate preparation, protein estimation, and immunoblot (IB) analysis

Lysates of Sertoli cells or testis were used for protein estimation and for IB analysis using corresponding antibodies (Table 1) as earlier described [20, 25]. For IB, an equal amount of total protein lysate between samples from Sertoli cells or testes at 30 μg protein were used. By spectrophotometry, protein estimation was performed using BCA Protein Assay Kit (KeyGEN Bio Tech Corp.; Cat#: KGP902). Protein signals in immunoblots were detected using Amersham Typhoon5 Biomolecular Imagers (GE Healthcare Life Sciences, Little Chalfont, UK). GAPDH, and/or β-actin served as protein loading controls. To avoid inter-experimental variations, all samples within an experimental group, including treatment and control samples, were analyzed simultaneously in a single experimental session.

### Microtubule (MT) spin-down assay to assess MT polymerization

The capability of MT polymerization in Sertoli cell lysates following Map1a RNAi *vs.* controls (cells transfected with non-targeting siRNA duplexes) was assessed by a spin-down assay using kits from Cytoskeleton (Denver, CO, USA; Cat# BK038) as earlier described [40, 41]. In brief, Sertoli cells obtained from the treatment *vs.* the control group were homogenized in 37 °C pre-warmed lysis and MT stabilization buffer (100 mM PIPES, 5 mM MgCl_2_, 1 mM EGTA, 0.1% NP-40, 0.1% Triton X-100, 0.1% Tween 20, 0.1% 2-mercaptoethanol, 30% glycerol, pH 6.9) to obtain lysates [40, 41]. Thereafter, lysates were centrifuged at 37 °C for 5 min at 2,000* g* to remove cellular debris, to be followed by centrifugation at 37 °C for 30 min at 100,000* g* to obtain polymerized tubulins (i.e., MTs) in the pellet, separated from free tubulin monomers of α- or ß-tubulin in the supernatant (S/N). Thereafter, the pellet (resuspended in 0.5 mM CaCl_2_ to a volume equal to the supernatant) and the supernatant were used for IB to quantify the relative level of polymerized tubulins. Incubation of Sertoli cell lysates with paclitaxel (3 μM, also known as Taxol, an MT stabilizing agent) *vs.* CaCl_2_ (0.5 mM, an MT depolymerization agent) served as the corresponding positive and negative controls, respectively. Assays were performed with *n* = 3 independent experiments and each experiment had triplicate Sertoli cell cultures.

### Assessing the relative F (filamentous) actin to G (globular) actin in Sertoli cell lysates following Map1a KD to assess relative actin polymerization

Changes in the relative level of F- and G-actin in Sertoli cell lysates following Map1a KD were performed using kits from Cytoskeleton (Denver, CO, USA; Cat# BK037) as described [42]. This assay assessed the ability of cell lysates from treatment *vs.* control cells to polymerize actin monomers (i.e., G-actin) into F-actin (in the pellet). In brief, Sertoli cell lysates were homogenized in F-actin stabilization buffer and pre-cleared by centrifugation at 350* g* for 5 min at room temperature, to be followed by centrifugation at 100.000* g* at 37 °C for 1 h to separate F-actin (pellet) from G-actin (in S/N). The supernatant containing G-actin was collected, whereas the pellet containing F-actin was dissolved in 80 mM urea. Both the supernatant and pellet of each sample were used for IB. Cell lysates treated with phalloidin (0.1 μM, an actin stabilizing agent) *vs.* urea (80 mM, an actin depolymerization agent) served as the corresponding positive and negative controls. Data in the control (control RNAi) were arbitrarily set at 1, against which data from samples of Map1a RNAi were statistically compared.

### Statistical analysis

Data analyses were performed using GraphPad Prism 8 software (GraphPad Software) with either Student’s *t*-test (for 2-group comparisons), or one-way analysis of variance (ANOVA) (for multi-group comparisons). Data presented are the mean ± SD of *n* = 3 to 5 independent experiments. *P* < 0.05 was considered statistically significant.

## Results

### Expression and distribution of Map1a in Sertoli cells in vitro, and its stage-specific distribution in the seminiferous epithelium of adult rat testes in vivo

**Fig. 1:**
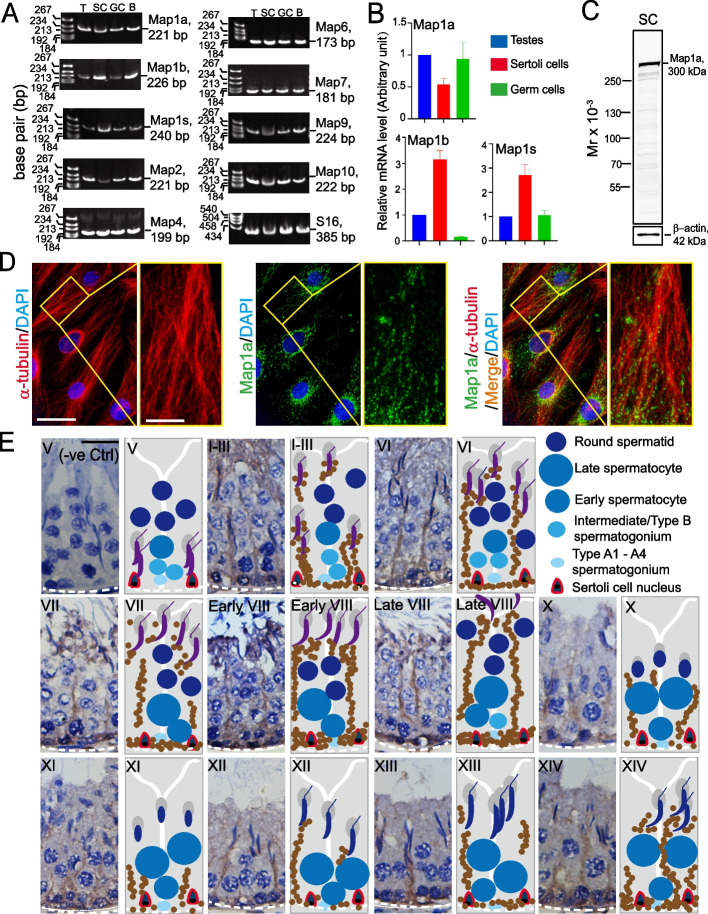
Identification of multiple microtubule-associated proteins (Maps) in rat testes and the distribution of Map1a in Sertoli cells and in rat testes. **A** RT-PCR was performed using corresponding primer pairs (Table 2) to examine the presence of multiple MAPs in adult rat testes *versus* Sertoli cells (SC) and germ cells (GC), with the brain (**B**) serving as a positive control. **B** qPCR was used to validate some data of RT-PCR as noted in (**A**). **C** The specificity of the anti-Map1a antibody (Table 1) was noted by IB using lysates of the corresponding cells or testis, with ß-actin serving as the protein loading control used for IF and IHC shown in (**D**) and (**E**), respectively. **D** IF (immunofluorescence analysis) illustrating the distribution of microtubules (visualized by α-tubulin staining, red fluorescence) and Map1a (green fluorescence) across the Sertoli cell cytoplasm (cell nuclei visualized by DAPI), as well as their co-localization. Scale bar, 40 µm; 15 µm in the enlarged image; which applies to corresponding images in this panel. **E** A study by IHC (immunohistochemistry) to illustrate changes in spatiotemporal and stage-specific expression of Map1a across the seminiferous epithelium during the epithelial cycle of spermatogenesis. Negative control (-ve Ctrl) is also noted on the *left* panel where the primary antibody was substituted with the IgG of the same animal species used to obtain the anti-Map1a antibody. Scale bar, 50 µm, applies to other images in this panel

**Fig. 2:**
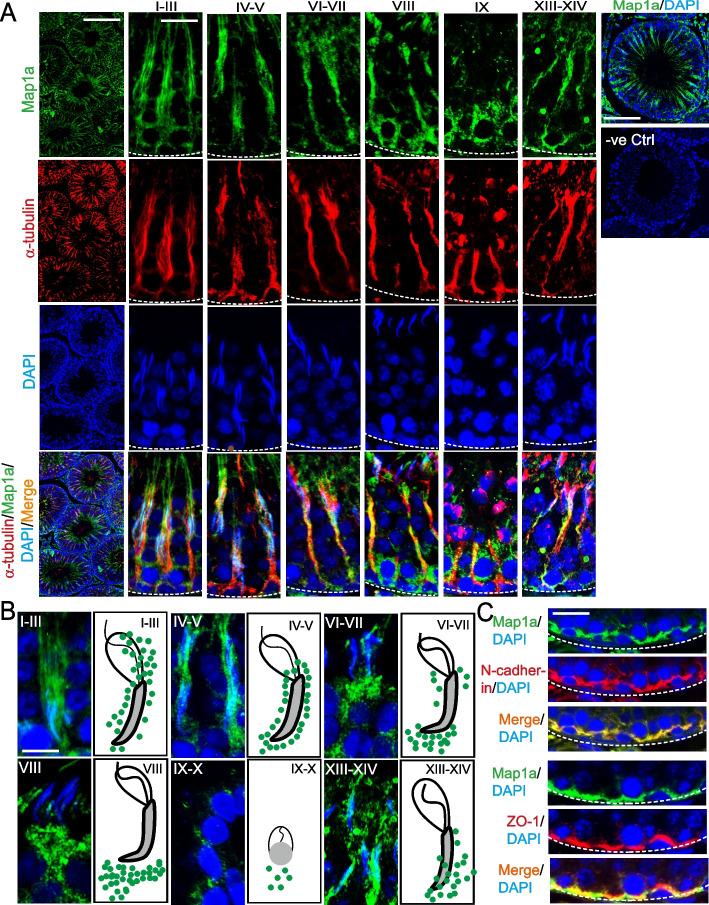
Stage-specific and spatiotemporal expression of Map1a across the seminiferous epithelium in adult rat testes during the epithelial cycle. **A** Map1a (green fluorescence) co-localized with microtubules (visualized by α-tubulin staining, red fluorescence), displaying stage-specific and spatiotemporal expression across the seminiferous epithelium. The right panel shows the results of a negative control when the primary antibody was substituted by using IgG of the corresponding animal species, with no detectable green fluorescence of Map1a was noted. Scale bar, 300 µm (left panel); 30 µm for the enlarged image; 100 µm in the right panel (control groups) to demonstrate no staining in negative control group. **B** Stage-specific expression and distribution at the apical ES with schematic drawing at the right panel. Scale bar, 20 µm, which applies to other images. **C** Co-localization of Map1a with basal ES (N-cadherin) and TJ (ZO-1) proteins at the BTB. Scale bar, 20 µm

Using RT-PCR (Fig. 1A) and qPCR (Fig. 1B) with primers specific to Map1a and selected members of its related Maps (Table 2), multiple Maps were shown to be expressed by Sertoli cells and germ cells in rat testes, with brain serving as a positive control (Fig. 1A, B). We elected to investigate Map1a in this report because its expression was relatively high in both Sertoli and germ cells based on RT-PCR (Fig. 1A) and qPCR (Fig. 1B); and specific antibodies again Map1a were commercially available (Table 1; Fig. 1C). Several anti-Map1a antibodies from different vendors were used in pilot experiments, these anti-Map1a antibodies listed in Table 1 were used for different experiments in our studies. It was found to stain an immunoreactive band by IB (immunoblotting) corresponding to the predicted apparent molecular weight (Mr) of Map1a at 300 kDa (Fig. 1C). Sertoli cells appeared to be the important source of Map1a in the testis, but germ cells also expressed a considerable amount of Map1a (Fig. 1A-C). Since Map1a is a regulatory protein known to support microtubule (MT) cytoskeletal dynamics by binding onto MT protofilaments to confer MT stabilization [3, 5, 43, 44], it is expected that Map1a should partially co-localize with MTs in Sertoli cells. Using immunofluorescence microscopy, MTs across the primary Sertoli cell cultured in vitro were visualized using a specific anti-α-tubulin antibody (Table 1), and α-tubulin together with ß-tubulin create the α-/ß-tubulin oligomers which are the building blocks of MTs (Fig. 1D). Consistent with earlier reports [20, 40], MT appeared as long stretches of protofilaments that laid across the entire Sertoli cell cytoplasm (red fluorescence) serving as tracks to support cellular organelles in cargo transports across the seminiferous epithelium in vivo (Fig. 1D). Whereas Map1a was noted as aggregates of green fluorescence dots that co-localized and laid along the track-like structures of microtubules, (Fig. 1D), consistent with its functional role in MT dynamics by binding onto microtubules [3, 5, 43, 44]. Map1a also displayed a prominent localization in the perinuclear cytoplasm of Sertoli cells (Fig. 1D) with an unknown function at this stage. Using IHC (immunohistochemistry), Map1a appeared as reddish-brown track-like structures (*left* panel) stretching across the entire seminiferous epithelium in the testis in vivo and changes in their distribution during the epithelial cycle were shown in the schematic drawings (*right* panel) in Fig. 1E). This pattern of expression noted by IHC was analogous to findings of dual-labeled immunofluorescence microscopy (Fig. 2A). In brief, Map1a co-localized with MTs across the seminiferous epithelium in the testis in vivo was shown in Fig. 2A. Collectively, these findings thus support the notion that Map1a is an MT stabilizing protein by binding onto MT protofilaments to maintain MT stabilization (Figs. 1E and 2A). Besides being closely associated with MT-based tracks across the epithelium, Map1a also localized prominently to the apical ES and the basal ES at the BTB, and with some staining at the tunica propria (Fig. 1E). Interestingly, its expression at the apical ES is stage-specific, being highly expressed at the apical ES, engulfing the entire elongated (condensed) spermatid head in stages I-VI tubules (Fig. 1E), and consistent with the data obtained by immunofluorescence microscopy (Fig. 2A, B). But Map1a no longer highly expressed across the entire spermatid head at the apical ES in stage VIII tubules (the only anchoring device in stage VIII tubules [45–47]) (Figs. 1E and 2A, B). Instead, Map1a expression was prominently at the tip of the spermatid head, and it appeared to be engulfed by Sertoli cells, and moved away from the spermatid head in late-stage VIII tubules as noted by IHC and IF in cross-sections of adult rat testes (Figs. 1E and 2A, B; see also the accompanying schematic drawings in Figs. 1E and 2B). Map1a considerably diminished in the apical ES in stages X, XI, and XII, but reappeared and associated with apical ES in stage XIII-XIV tubules (Figs. 1E and 2A, B). For basal ES/BTB, Map1a notably expressed at this site in stages I-VII but most intensely in stage VIII tubules, its expression declined in stages X-XII, but it began to express considerably by stages XIII-XIV (Fig. 1E and 2A). Map1a also co-localized with putative basal ES protein N-cadherin and TJ protein ZO-1 in adult rat testes (Fig. 2C). It is of interest that Map1a was notably expressed in testicular germ cells (Fig. 1B), but in Figs. 1E and 2A, Map1a was prominently associated with MT-based tracks across the seminiferous epithelium. However, distinctive Map1a was found near the base of the seminiferous epithelium, close to the basement membrane of the tunica propria, appearing to be expressed by late pachytene spermatocytes, most notably in stage X-XIV tubules (Figs. 1E and 2A). But its function in these germ cells remains unknown.

### Expression and localization of Map1a across the seminiferous epithelium during CdC_2_-induced testis injury

**Fig. 3:**
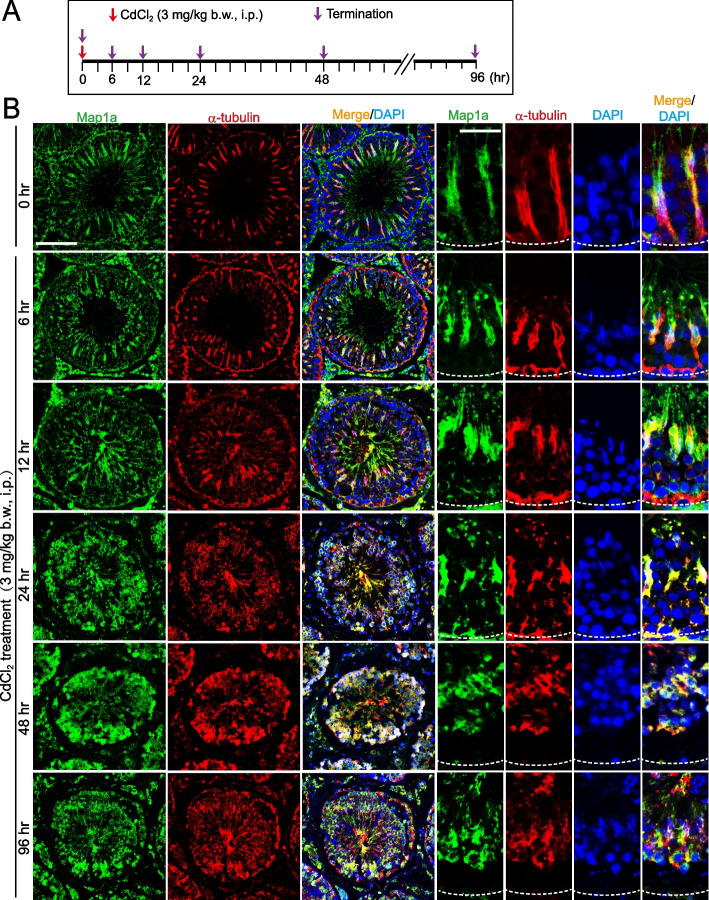
Changes in spatiotemporal expression of Map1a and microtubule organization during cadmium-induced testis injury. **A** The regiment used for the study of a CdCl_2_-induced testis injury animal model with *n* = 4 adult rats at each time point. CdCl_2_, was dissolved in saline at 20 mg/ml and administered to each adult rat (~ 300 gm b.w.) by intra-peritoneal (ip) injection using a 28-gauge needle syringe at a dose of 3 mg/kg b.w. **B** Map1a (green fluorescence) appeared as track-like structures that lay perpendicular to the basement membrane (annotated by dotted white line) near the base of a seminiferous tubule. Map1a stretched across the entire epithelium, co-localized with microtubules (visualized by α-tubulin staining, red fluorescence, see also merge image). MTs were truncated, possibly due to the mis-localization of Map1a, which no longer capable of promoting MT stabilization, thereby leading to microtubule catastrophe. Scale bar, 100 µm; 30 µm in magnified images, which apply to corresponding images

**Fig. 4:**
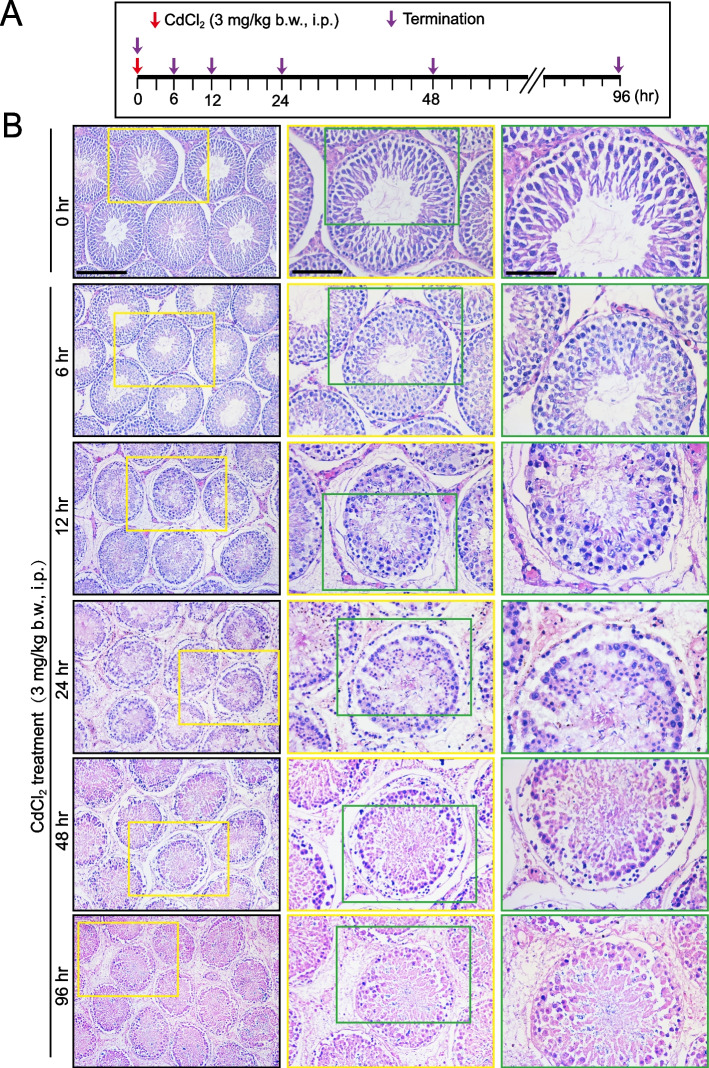
Changes in the seminiferous epithelial architecture during CdCl_2_-induced testis injury. **A** The regimen used for the experiments reported in (**B**). **B** Changes in the architectural organization of the seminiferous epithelium during CdCl_2_-induced testis injury time-dependently with gross epithelial structural damage detected by as early as 12 h. These damages include a time-dependent reduction in tubule diameter, a considerable loss of tubule lumen space due to exfoliation of germ cells, appearance of vacuoles across the epithelium, an indicator of Sertoli cell injury [53, 55, 57]. This figure was prepared using a representative set of adult rats (~ 300 gm b.w.) and two other animal sets yielded similar results. Scale bar, 300 µm, 100 µm, and 50 µm, corresponding to the first image of the first, second, and third columns in the *top* panel

Since Map1a is tightly associated with MTs across the seminiferous epithelium to support MT cytoskeletal function as noted in Figs. 1 and 2, we sought to use an animal model of cadmium-induced testis injury which is known to exert its disruptive effects to impair reproductive function [20, 45, 48–51] through changes on cytoskeletons [31, 52] to examine the functional relationship of Map1a and microtubules. Using adult rats and the regimen shown in Fig. 3A, CdCl_2_ (3 mg/kg b.w., i.p.) was found to induce rapid disorganization of the MT cytoskeleton as early as 6 h (Fig. 3B). Within 6 h following CdCl_2_ treatment, MTs no longer stretched as linear tracks that aligned perpendicular to the basement membrane (annotated by dashed white line) and stretched across the entire seminiferous epithelium as noted in control testes (Fig. 3B). Instead, MTs in CdC_2_-treated testes appeared as truncated fragments, which were broken down into very short fragments and randomly aligned across the entire epithelium (Fig. 3B). This pattern of changes in the disruptive organization of MTs mimicked by similar disruptive changes regarding the temporal and spatial expression of Map1a across the epithelium (Fig. 3B). As such, MTs no longer served as the functional dynamic linear structures across the seminiferous epithelium but undergoing catastrophes as noted in Fig. 3B due to the disruptive temporal and spatial expression of Map1a (Fig. 3B). These changes thus led to morphological disruption across the seminiferous epithelium as noted in the histological data shown in Fig. 4. It was using the regimen noted in Fig. 4A, treatment of testes by CdCl_2_ led to gross morphological changes including seminiferous epithelial atrophy in which tubules were shrunk by as much as 50% within 96 h with distinctive large vacuoles developed across the epithelium, which were earlier shown to be typical features of Sertoli cell injury [53–55]. These findings are also consistent with earlier reports that the MT cytoskeleton is one of the primary targets of environmental toxicants [48, 56].

### Map1a knockdown (KD) by RNAi perturbs the Sertoli cell TJ barrier due to the disruptive distribution of TJ and basal ES proteins

**Fig. 5:**
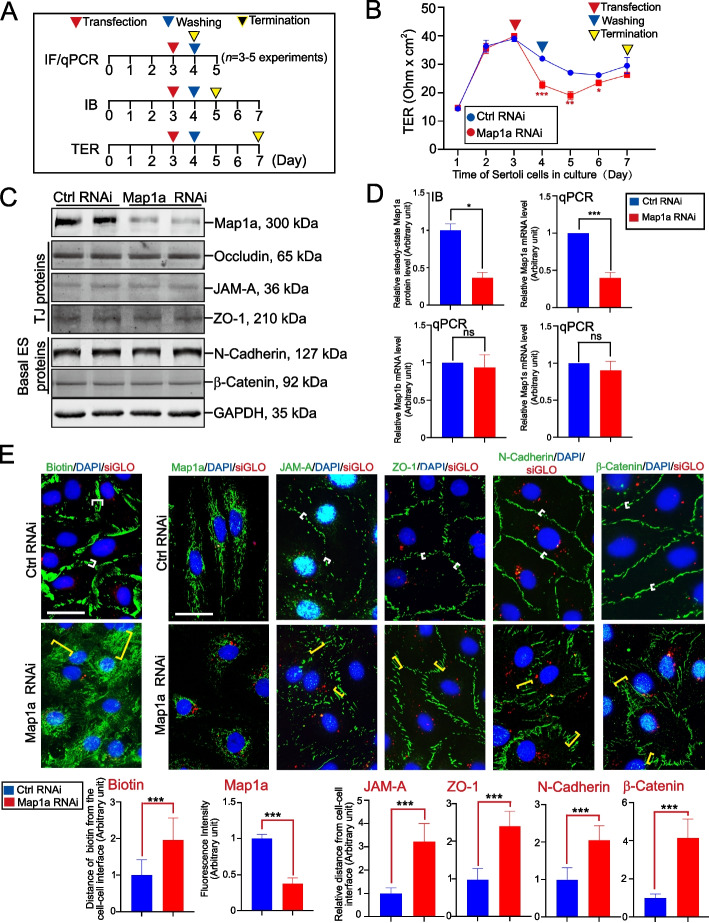
Map1a knockdown (KD) in Sertoli cells by RNAi perturbs the Sertoli cell TJ-permeability function due to changes in the distribution of TJ and basal ES proteins at the BTB. **A** The regimen used for the studies reported in Figs. 5, 6, and 7. **B** Following the establishment of a functional Sertoli TJ-barrier (manifested by the presence of stable TER across the cell epithelium cultured on Matrigel-coated bicameral units, see *Materials and Methods*), Map1a KD was found to perturb the Sertoli cell TJ-barrier after cells were transfected with the Map1a-specific siRNA duplexes *versus* non-targeting control siRNA duplexes (Table 3). Each data point is a mean ± SD of quadruple bicameral units from a representative experiment, from a total of *n* = 4 independent experiments using different batches of Sertoli cells, which yielded similar results. *, *P* < 0.05; **, *P* < 0.01; ***, *P* < 0.005, by paired Student’s *t*-test, compared to the corresponding control. **C** A study by IB analysis confirmed the KD of Map1a expression without affecting the expression of other TJ- and basal ES-proteins at the Sertoli cell BTB. **D** Composite data of bar graphs of IB results to illustrate Map1a KD with *n* = 3 experiments. qPCR results confirmed the KD of Map1a without affecting the expression of Map1b and Map1s using primer pairs specific to the corresponding genes (Table 3). **E** Results of an in vitro BTB functional assay, illustrating KD of Map1a led to a disruption of the Sertoli BTB, incapable of blocking the entry of a small membrane impermeable biotin (green fluorescence) into the cell cytoplasm, unlike the control Sertoli cells (*left* panel). KD of Map1a was noted by IF analysis. KD of Map1a led to mis-distribution of TJ (JAM-A and ZO-1) and basal ES (N-cadherin, ß-catenin) proteins, no longer tightly localized at the Sertoli cell–cell interface to support BTB function, instead these proteins were diffusely localized, leading to BTB disruption as noted in (**B**) and (**E**, *left* panel). Composite semi-quantitative data of the *top* panel are noted in the bar graphs with each bar represents a mean ± SD of *n* = 3 experiments. At least 50 randomly Sertoli cell pairs were analyzed from each experiment from a total of *n* = 3 experiments

The regimen used for the study reported herein is noted in Fig. 5A. Primary Sertoli cells cultured in vitro were capable of forming a functional TJ barrier that mimicked the Sertoli cell BTB in vivo [26, 58–60] within ~ 2–3 days in serum-free chemically defined F12/DMEM as noted by a stable permeability barrier (Fig. 5B). However, transfection of the intact Sertoli cell epithelium on day 3 with an established functional TJ-barrier as manifested by a stable TER with Map1a siRNA duplexes *versus* non-targeting control (Ctrl) siRNA duplexes (Table 3) for 24 h led to a disruption of the permeability barrier (Fig. 5B). But the disrupted barrier was resealed by day 7, illustrating the RNAi-induced TJ disruption was transient and reversible. The efficacy of Map1a silencing was noted by IB (Fig. 5C), without affecting a number of structural TJ (e.g., occludin, JAM-A, and ZO-1) and basal ES (e.g., N-cadherin, ß-catenin) proteins (Fig. 5C), but also signaling proteins and PCP proteins that were found at the Sertoli cell BTB, and were earlier shown to modulate Sertoli cell function [61, 62] (Figures S1, S2). Composite data on the IB of Map1a (Fig. 5C) illustrated a ~ 75% KD of Map1a by RNAi (Fig. 5D, *top left* panel), consistent with the results of qPCR (Fig. 5D*, top right* panel). Since antibodies against Map1b and Map1s were not commercially available for IB, qPCR was used to verify that Map1b and Map1s were unaffected (Fig. 5D, *lower* panels). Collectively, data shown in Figs. 5C, D; and Figures S1, S2 and S3, thus support the notion that there were no off-target effects of Map1a in these RNAi experiments. The primer pairs used for qPCR are noted in Table 2. Using immunofluorescence microscopy and a functional in vitro assay to monitor the integrity of the Sertoli cell TJ-barrier by blocking the diffusion of biotin across the permeability barrier as reported earlier [36, 37], Map1a KD by RNAi effectively made the BTB “leaky” as noted in this functional assay when compared to the control group (Fig. 5E, *left* panel). The effectiveness of RNAi in silencing Map1a expression was also confirmed by fluorescent microscopy (Fig. 5E, see also composite data in the lower panel). Disruptive changes in the distribution of TJ (e.g., JAM-A, ZO-1) and basal ES (e.g., N-cadherin, ß-catenin) proteins at the Sertoli cell–cell interface wherein these proteins at the BTB no longer tightly associated with the TJ/basal ES site but diffusely localized (Fig. 5E), thereby disrupting the TJ-barrier as noted in Fig. 5B.

### Map1a KD by RNAi perturbs the organization of Sertoli cell microtubules by impeding MT polymerization

**Fig. 6:**
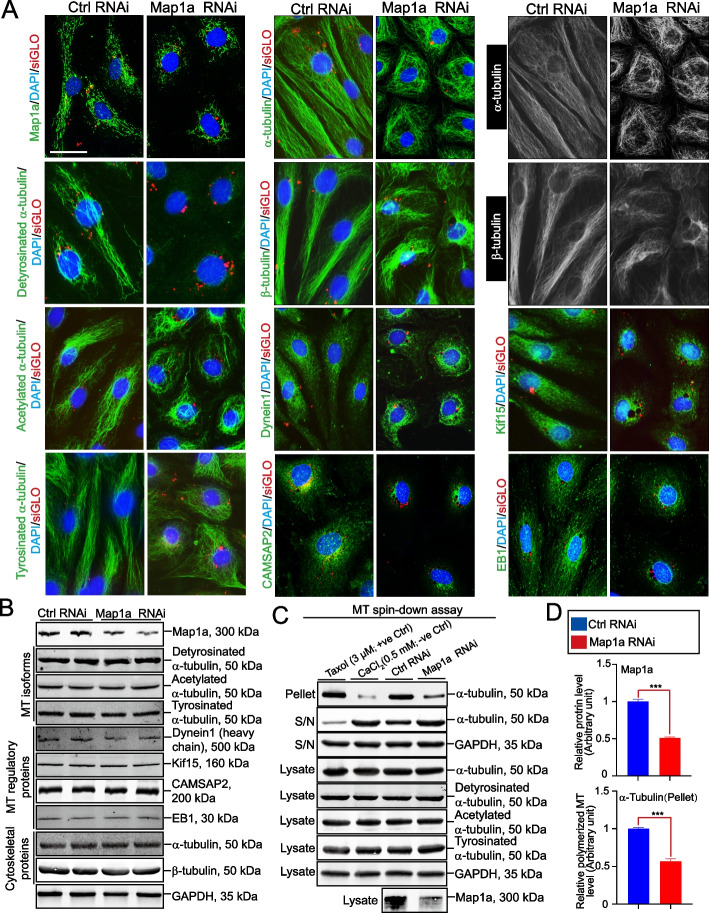
Map1a KD perturbs microtubule (MT) organization through disruption of MT polymerization. **A** Using IF analysis, effective KD of Map1a (*top left* panel) perturbed the organization of MTs across the Sertoli cell cytoplasm since microtubules no longer stretched across the entire cell cytoplasm as noted in control cells, but retracted from cell peripheries and wrapped around the cell nuclei. Such changes were noted in the isoforms of MTs, including detyrosinated α-tubulin and acetylated α-tubulin, representing the stabilized forms of MTs; and tyrosinated α-tubulin (the dynamic, un-stabilized form of MTs). The distribution of dynein 1, CAMSAP2, Kif15, and EB1 were also perturbed following Map1a KD. Scale bar, 30 µm, which applies to other micrographs. **B** IB analysis illustrates the effectiveness of Map1a KD, without affecting other MT isoforms, MT regulatory proteins and cytoskeletal proteins after Map1a KD. **C** Results of a biochemical assay that monitored MT polymerization activity of the Sertoli cell lysates from Map1a KD cells *versus* control cells. Importantly, the effective KD of Map1a (see bottom panel) did not affect the expression of the MT isoforms, but it considerably inhibited MT polymerization. **D** Composite data of the MT polymerization assay illustrates the effective KD of Map1a that led to a considerable disruption of MT polymerization. ***, *P* < 0.005, by paired Student's *t*-test, compared to the corresponding control (Ctrl)

Using the regimen noted in Fig. 5A, we next examined whether Map1a KD affected Sertoli cell MT organization. First, Map1a KD effectively reduced the expression of Map1a in Sertoli cells (Fig. 6A, *top left* panel). In control groups, wherein Sertoli cells were transfected with non-targeting control siRNA duplexes, detyrosinated and acetylated α-tubulin (both are isoforms of MTs used to stabilize MTs) [63], and tyrosinated α-tubulin (to promote MT dynamics, making MTs less stable) [63, 64], similar to α-tubulin and ß-tubulin, stretched across the Sertoli cell cytosol as linear structures (Fig. 6A). Yet Map1a RNAi rendered these microtubule protofilaments to become fragmented and wrapped around the Sertoli cell nuclei, retracting from the cell peripheries (Fig. 6A). Considerable and distinctive changes in the distribution of dynein 1 (an MT-dependent minus-end directed motor protein) [17], CAMSAP2 (an MT minus-end targeting protein, -TIP) [65], Kif15 (an MT-dependent plus-end directed motor protein) [18] and EB1 (an MT plus-end tracking protein, + TIP) [40] were also noted following Map1a KD when compared to control cells, which, in turn, impeded MT cytoskeletal organization (Fig. 6A). While Map1a RNAi silenced the expression of Map1a, it did not affect the expression of any MT isoforms nor the MT-regulatory proteins, namely dynein 1, Kif15, CAMSAP2, and EB1 as noted by IB analysis (Fig. 6B, Figure S4). More importantly, Map1a KD also considerably reduced the ability of MT polymerization based on the use of biochemical assay following Map1a KD as noted in Figs. 6C, D, Figure S5, with the use of Taxol (3 µM) and CaCl_2_ (0.5 mM) served as the corresponding positive and negative controls (Fig. 6C).

### Map1a KD by RNAi in Sertoli cells impedes actin cytoskeletal organization and actin polymerization and bundling capability

**Fig. 7:**
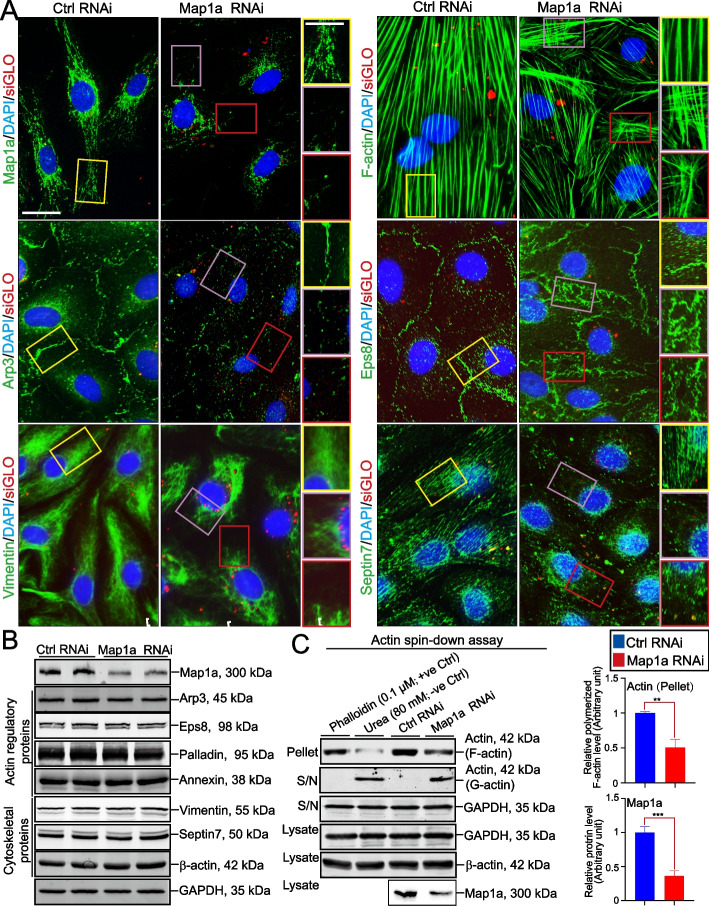
Map1a KD perturbs actin organization through disruption of actin polymerization and filament bundling activity. **A** Effective KD of Map1a as noted by IF analysis led to the truncation of actin filaments across the Sertoli cell cytoplasm, distinctively different from control Sertoli cells. These changes appeared to be the result of the disruptive distribution of Arp3 (a branched actin polymerization-inducing protein) and Eps8 (an actin barbed-end capping and bundling protein) in Sertoli cells. Also, vimentin-based intermediate filaments and septin7-based cytoskeleton were also grossly perturbed in Map1a silenced cells *versus* control cells. Data shown here are representative findings from an experiment and *n* = 3 experiments yielded similar results. Scale bar, 20 µm, and 15 µm in inset; which apply to other corresponding micrographs in the same panel. **B** Effective KD of Map1a in Sertoli cells as noted by IB analysis did not affect the steady-state protein levels of several actin regulator proteins and other structural/cytoskeletal proteins, supporting the notion that there were no off-target effects of Map1a KD. **C** Using a biochemical assay to assess actin polymerization and actin-bundling activity (F-actin), Map1a considerably perturbed actin polymerization and the formation of F-actin. Composite data of these findings are shown in the bar graphs on the *right* panel that illustrate that Map1a KD led to effective disruption of actin polymerization. Each bar is a mean ± SD of *n* = 3 experiments. **, *P* < 0.01; ***, *P* < 0.005 by paired Student’s *t*-test

Using the regimen of Fig. 5A, we also examined if Map1a KD that perturbed Sertoli cell TJ-barrier function mediated its effects through actin cytoskeletal organization. Effective KD of Map1a expression (Fig. 7A, B, C) was found to perturb actin filament organization across the Sertoli cell cytoplasm wherein linear actin filaments became truncated (Fig. 7A). Such disorganization of the actin cytoskeleton across the Sertoli cell might be the result of the disruptive distribution of Arp3 (which together with Arp2 creates the Arp2/3 complex when activated with N-WASP was shown to induce branched actin polymerization [66]) and Eps8 (an actin barbed-end capping and bundling protein [66]) in the Sertoli cell epithelium (Fig. 7A). This, in turn, perturbed the actin cytoskeletal organization. Interestingly, Map1a KD also led to the disruptive organization of a vimentin-based intermediate filament network and septin7-based cytoskeleton in Sertoli cells, grossly different from control Sertoli cells (Fig. 7A). Also, the steady-state levels of these regulatory and structural proteins did not change after Map1a KD based on IB analysis (Figs. 7B, S6). However, the ability of Sertoli cell lysates to polymerize actin filaments to form F-actin was considerably perturbed (Figs. 7C, S7, see also composite data on the *right* panel). In this biochemical assay, phalloidin (0.1 µM) and urea (80 mM) served as the corresponding positive and negative controls which are known to promote and inhibit actin polymerization, respectively, thereby supporting the validity of this assay (Fig. 7C, S7). Composite data from *n* = 3 experiments were noted in the bar graph on the *right* panel (Fig. 7C). The findings in Fig. 7A-C also support the notion that Map1a is involved in supporting actin polymerization and bundling by conferring the actin filament bundles at the ES, perhaps through the stabilization of the adjacent MT network that promotes ES network as these two cytoskeletons are lying adjacent to one another and functionally connected [8, 67].

### Down-regulation of Map1a in the testis in vivo and Disruptive Distribution of Map1a in Sertoli cells in vitro induced by CdCl_2_ treatment lead to MT disorganization

**Fig. 8:**
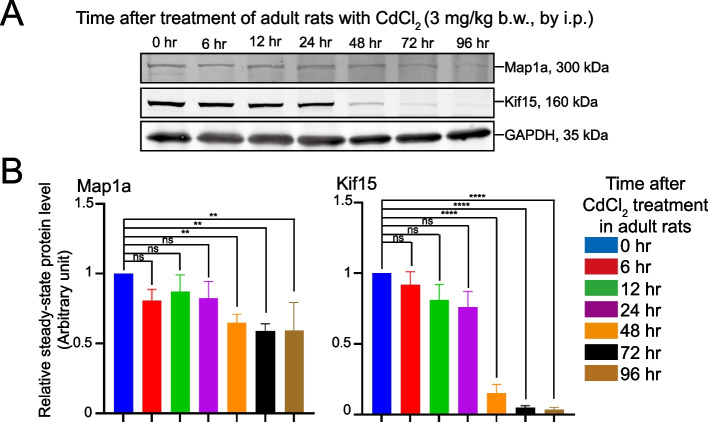
CdCl_2_ down-regulates expression of microtubule-associated proteins (Maps) in testes of adult rats. **A** Adult rats at 250–280 gm b.w. in groups of *n* = 3 rats were treated with a single dose of CdCl_2_ (3 mg/kg b.w., by intraperitoneal, i.p., administration) as described [33, 73, 74] at time 0 h (control, Ctrl), and then terminated at specified time points at 6, 12, 24, 48, 72, and 96 h. One testis was used for histological and IF analyses, and the other testis for IB. As such, each reported experiment had *n* = 3 testes from different rats for analysis. Lysates were obtained from these rats for IB analysis. A time-dependent down-regulation of Map1a and Kif15 was noted, wherein GAPDH served as a protein loading control. Results of a representation experiment were shown from *n* = 3 independent experiments. **B** Results of composite data with each bar representing a mean ± SD of *n* = 3 experiments on two Maps namely Map1a and Kif15 (a microtubule-dependent plus-end directed motor protein that moves cargoes towards the plus ( +)-end of the polarized MT protofilaments. **, *P* <0.01; ***, *P* < 0.005 by paired Student's *t*-test; ns, non-significantly

**Fig. 9:**
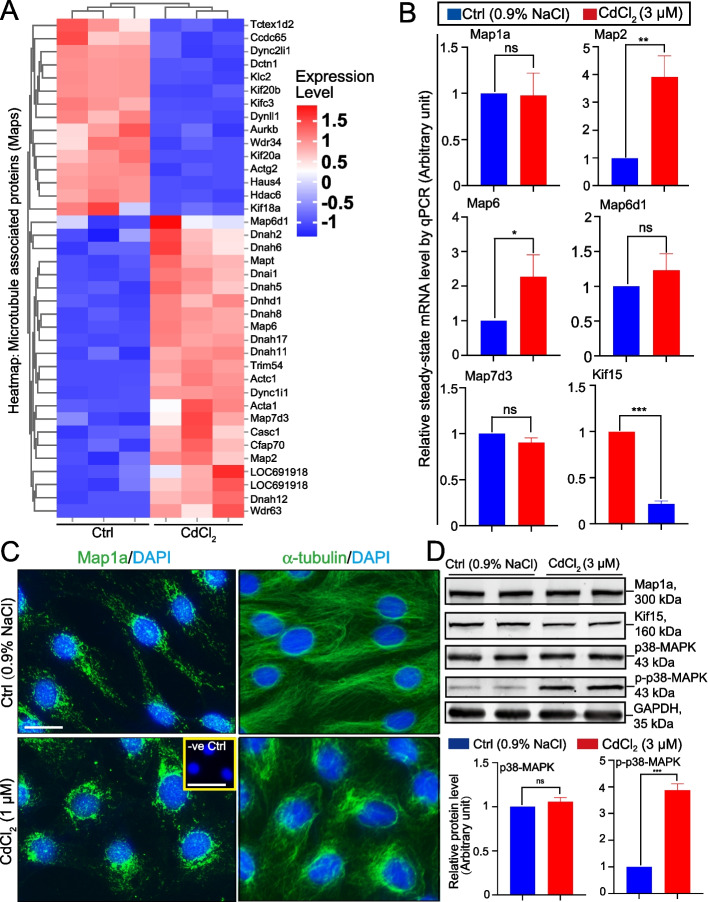
CdCl_2_ treatment of Sertoli cells in vitro induces changes in the expression of multiple Maps. Sertoli cells treated with CdCl_2_ (3 µM) vs. control (Ctrl, 0.9% saline) for 24 h were used for bulk RNA-Seq, qPCR, and immunoblot (IB) analysis. For IF analysis, CdCl_2_ at 1 µM for 6 h incubation was used. **A** Heatmap analysis of Maps illustrating multiple genes of MAPs that were either up (red) or down (blue) regulated compared between control (Ctrl) and CdCl_2_ treated Sertoli cell groups. These data were from *n* = 6 independent experiments used for bulk RNA-Seq which have been deposited at NCBI as Sequence Read Archive data (see *Materials and Methods*) as reported [25]. **B** qPCR data showing results of different Maps of *n* = 3 experiments in which the control (Ctrl) was arbitrarily set at 1 against which statistical analysis of paired Student’s *t*-test was performed. *, *P* < 0.05; **, *P* < 0.01; ***, *P* < 0.005; ns, not significantly different. **C** Distribution of Map1a across Sertoli cells was examined by IF in which Map1a appeared as aggregates along the protofilaments of microtubules that stretched across the entire Sertoli cell cytoplasm, consistent with the reported function of Map1a by binding onto the microtubule protofilaments to promote MT stabilization. For -ve Ctrl (negative control), the primary antibody against Map1a was substituted by the corresponding IgG of the same animal species. Scale bar, 20 µm; 200 µm in inset. Results shown here are representative findings of a single experiment from *n* = 3 independent experiments which yielded similar results. **D** IB analysis showing negligible changes in the protein expression of Map1a, but relatively mild down-regulation of Kif15, after CdCl_2_ (3 µM) treatment for 24 h. These IB data are consistent with findings shown in Fig. 8 since notable changes in the expression of these proteins were detected 48 and 72 h after CdCl_2_ treatment. Interestingly, CdCl_2_ induced a considerable up-regulation of p-p38-MAPK but not total p38-MAPK. The bar graph in the *lower* panel are composite data of IBs shown in the *upper* panel with each bar representing a mean ± SD of *n* = 3 experiments. Each experiment has triplicate cultures. ***, *P* < 0.005 by paired Student's *t*-test; ns, non-significantly different

As reported herein, Map1a KD down-regulated Map1a expression by ~ 70% was found to induce cytoskeletal disorganization of both actin and microtubule network, consistent with the physiological function of Map1a to promote MT stability [3, 5, 68]. This transient loss of MT stability can in turn impede actin network organization since actin cytoskeleton homeostasis requires optimal intracellular transport of cargoes along the MT-based tracks [69–71]. To provide better mechanistic insights on how Map1a modulates MT dynamics, we opted to use a toxicant model for our study. As environmental toxicant CdCl_2_ is known to induce Sertoli cell and testis injury through disruptive changes in the actin and MT cytoskeletons [16, 45, 48, 72], we sought to examine if rats were treated with CdCl_2_ (3 mg/kg b.w.) in vivo that affected cytoskeletal organization (see Fig. 3) would down-regulate Map1a expression. Indeed, CdCl_2_ down-regulated Map1a expression in rat testes time-dependently following CdCl_2_ administration (i.p.) and its effect was most notable by 72 and 96 h post-CdCl_2_ treatment (Fig. 8A, B; Figure S8A). Interestingly, another Map named Kif15, an MT-dependent plus-end directed motor protein recently studied in our laboratory and shown to support cargo transport [18], was also considerably down-regulated in the testis following treatment of rats with CdCl_2_ in vivo (Fig. 8A, B; Figure S8A). We next isolated RNAs from these Sertoli cells for bulk RNA-Seq for bioinformatics analysis to identify if there were any changes in signaling cascades using the primary Sertoli cell culture model. As noted in the Heatmap shown in Fig. 9A, a considerable number of Maps were either up- or down-regulated during CdCl_2_-induced Sertoli cell injury (Fig. 9A, B). While the use of qPCR confirmed the considerable down-regulation of Kif15 (Fig. 9B), Map1a was not considerably affected (Fig. 9B). Interestingly, CdCl_2_ notably perturbed the distribution of Map1a across the Sertoli cell cytoplasm within 24 h, making Map1a no longer distributed evenly across the protofilaments of the MT network (Fig. 9C, *left* panel) when the MT cytoskeletal network was grossly affected (Fig. 9C, *right* panel), possibly the result of mis-distribution of Map1a to promote MT stability. IB data shown in Fig. 9D (Figure S8B) are consistent with the in vivo data in Fig. 8. For instance, as early as 24 h, changes in the steady-state protein level of Map1a were not notably detected, *even* though a mild down-regulation of Kif 15 was observed (Figs. 9D, S8B) consistent with qPCR data shown in Fig. 9B. Interestingly, the steady-state level of p-p38-MAPK was considerably up-regulated but the total p38-MAPK was not affected (see also composite data of bar graph in the *lower* panel of Fig. 9D), suggesting the involvement of p-p38-MAPK activation during cadmium-induced changes in Map1a expression.

### Cadmium-induced Sertoli cell MT cytoskeletal dis-organization is possibly mediated through p-38 MAPK

**Fig. 10:**
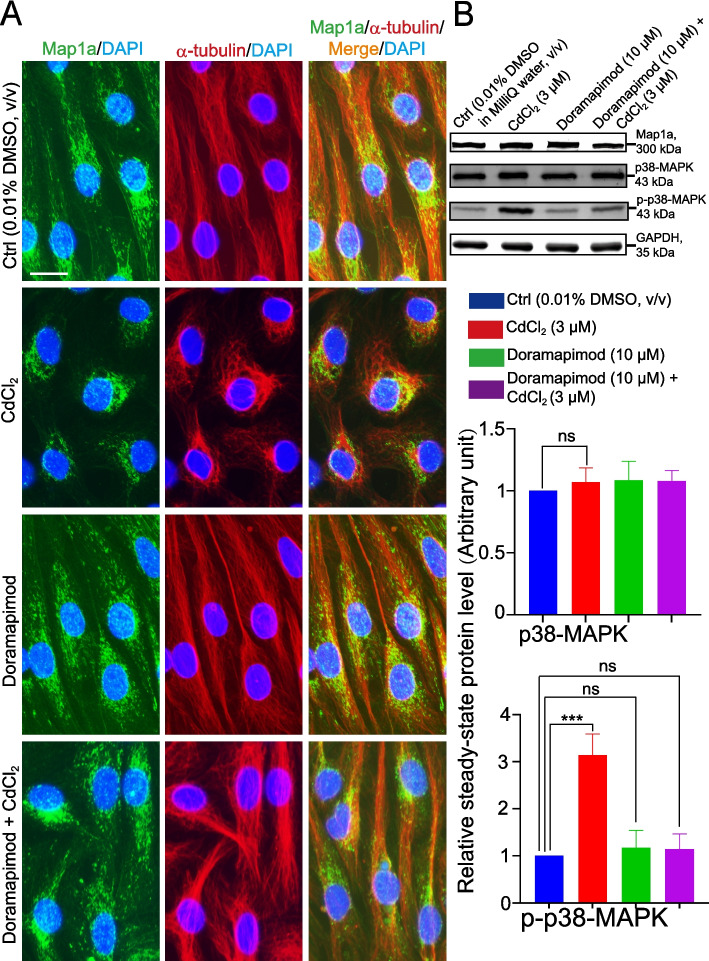
Cadmium-induced Sertoli cell injury that affects the cytoskeletal organization of microtubules is mediated through p38 MAPK activation. **A** A study by immunofluorescence microscopy to assess the organization of microtubule cytoskeleton network across the Sertoli cell cytoplasm. Pre-treatment of Sertoli cell epithelium on day 3 with doramapimod (10 µM), a p38 MAPK inhibitor, was able to block the cadmium-induced Sertoli cell injury by causing dis-organization of the MT network across the cell cytoplasm. CdCl_2_ dissolved in 0.9% saline (v/v, in Milli Q water) was used at 1 µM. Results shown here are representative findings of an experiment from *n* = 3 independent experiments using different batches of Sertoli cells which yielded similar observations. Scale bar, 20 µm, which applies to other micrographs in this panel. **B** Representative IBs from *n* = 3 experiments with GAPDH served as the protein loading control (*top* panel). Composite data are shown in the *lower* panel, with each bar representing a mean ± SD of *n* = 3 experiments, illustrating that treatment of Sertoli cells with CdCl_2_ at 3 µM for 24 h did not induce changes in Map1a expression even though its distribution across the cell cytoplasm was grossly perturbed as noted in A. However, p-p38-MAPK was induced by CdCl_2_, and its up-regulation was blocked by doramapimod. Each sample had triplicate cultures in each experiment. ***, *P* < 0.005; ns, non-significantly different

A recent report using the cadmium model has shown that cadmium-induced Sertoli cell dysfunction by perturbing the tight junction permeability barrier function is mediated via the p38 MAPK signaling pathway [25]. This conclusion was reached based on the use of a selective inhibitor named doramapimod (10 µM), which was capable of blocking cadmium-induced Sertoli cell injury [25] as manifested by changes in the tight junction permeability barrier function. However, the use of doramapimod was able to “re-seal” the cadmium-induced TJ “leaky” barrier [25]. As noted in Fig. 10A, pre-treatment of Sertoli cells with doramapimod indeed was able to block the cadmium-induced MT disorganization across the Sertoli cell cytoplasm, but doramapimod alone had no apparent effects (Fig. 10A). These findings noted in Fig. 10A thus support the notion that cadmium-induced Sertoli cell injury that perturbed the MT network through changes in Map1a distribution across the Sertoli cells was mediated via p-38 MAPK activation. Indeed, this conclusion was also supported by the IB data shown in Fig. 10B and Figure S8C, illustrating doramapimod indeed blocked the activation of p-p38 MAPK and that the use of doramapimod was capable of blocking the cadmium-induced up-regulation of p-p38 MAPK (Fig. 10B; see also composite data in the bar graph in the *lower* panel). In brief, doramapimod thus rescued the cadmium-induced cytoskeletal disorganization of microtubules across the Sertoli cell cytoplasm, illustrating the involvement of p-p38-MAPK signaling in cadmium-induced Sertoli cell injury mediated through changes in the distribution of Map1a, which in turn affected MT organization due to its intrinsic activity on MT stabilization.

## Discussion

Microtubule-associated proteins (Maps), such as Map1a, play crucial roles in supporting microtubule functions in mammalian cells which in turn support other cellular functions. These include intracellular organelle transports, cell divisions, cell shape/organization, cell polarity, cell movement, cell differentiation, pathogenesis, and others [1, 70, 75–77]. In testes, the microtubule (MT) cytoskeleton works in concert with the actin cytoskeleton in Sertoli cells to support intracellular protein/organelle trafficking but also spermatogenesis, most notably, germ cell development and the progressive movement of developing haploid spermatids, across the seminiferous epithelium during the epithelial cycle of spermatogenesis in both rodents and humans [8, 45, 78–82]. The MT and the actin cytoskeletons are particularly unique in the testis of rodents and humans since developing haploid spermatids are virtually metabolically quiescence cells during spermiogenesis with minimal cytoplasm (almost none exist in late-stage condensed spermatids), unlike other mammalian cells, by committing all of their resources in three key cellular processes. First, by re-organizing and compacting their chromatin material to become the condensed nucleus in the spermatid head with virtually no transcription activity thereafter [83–87]. Second, the acrosome is formed by the vacuoles of the Golgi body by filling with lytic enzymes (e.g., acrosin) and forming a cap-like structure at the anterior end of the spermatid head [88, 89]. Third, assembly of the mid-piece of the tail with tightly packed mitochondria to provide the ATPs necessary to support the flagellar movement of the sperm tails following the maturation of sperms in the epididymis [90, 91]. The conventional concept is that these cytoskeletons are to be used for intracellular trafficking supported by corresponding actin- or MT-dependent plus ( +) or minus (-) end motor proteins. Since developing haploid spermatids reside outside the Sertoli cells near the base of the seminiferous tubule without any notable cell motility-dependent ultra-structures such as lamellipodia and filopodia, how could these spermatids migrate from the basal to the adluminal compartment near the tubule n during spermiogenesis? Studies in the 1970s and 1980s have noted that there is a unique structure at the Sertoli cell-spermatid interface designated ectoplasmic specialization (ES) [92, 93]. At the ES, the plasma membrane of step 8 spermatids (and beyond) in rodents becomes tightly anchored onto the Sertoli cell plasma membrane, persisting throughout spermiogenesis, and transforming into a transient structure called tubulobulbar complex [94, 95] to support the subsequent release of sperms at spermiation [46, 96]. It is notated that at the ES, spermatids reside outside the Sertoli cell plasma membrane but they were tightly anchored onto the Sertoli cell MT track-like structure located near the plasma membrane [8, 67]. The MT-dependent minus-end directed motor proteins (e.g., dynein 1) [17] or the MT-dependent plus-end directed motor proteins (e.g., kinesin 15) [18] can thus “transport” (or “push”) the spermatids that are tightly anchored onto the Sertoli-spermatid interface towards either the tubule lumen (at the MT minus end) at stage VIII or the base of the tubule (at the MT plus end) near the basement membrane at stage V of the epithelial cycle in the rat testis [47]. This is analogous to an intracellular “cargo” transport within the Sertoli cell cytoplasm even though spermatids reside outside the Sertoli cell through the unique ES ultrastructure. This directional transport of spermatids across the seminiferous epithelium is supported by the polarized microtubule-based tracks, which, in turn, are conferred by either the + TIPs (e.g., EB1) [40] or the -TIPs (e.g., CAMSAP2) [65] as recently reported. In brief, the testis utilizes a specialized cell ultrastructure designated ES to support spermatid transport outside the Sertoli cell plasma membrane, eliminating the necessity of expressing cell motility ultra-structures of actin- and MT-dependent lamellipodia and filopodia as found in other motile cells. It is because of this unique spermatid transport mechanism, that the regulation of MT dynamics is crucial to understanding the biology of spermiogenesis during spermatogenesis.

To the best of our knowledge, there are virtually no detailed functional studies in the literature regarding the role of Maps in modulating MT function. Interestingly, multiple Maps are detected in adult rat testes, expressed by both Sertoli and germ cells, most likely pre-meiotic germ cells. We elected Map1a for our studies as reported here because it is one of the predominant Maps and we were able to identify a suitable antibody for our studies. The notable features are that Map1a co-localized with MTs across the Sertoli cell cytoplasmic. Using the cross-sections of testes for studies in vivo, Map1a displays stage-specific expression across the seminiferous epithelium. Map1a co-localizes with MTs by aligning perpendicular to the basement membrane and stretching across the entire seminiferous epithelium as track-like structures. More importantly, Map1a mis-distribution mimics the cadmium-induced MT disorganization, which becomes extensively fragmented within 6 to 24 h after CdCl_2_ treatment. These changes lead to gross epithelial damage such as exfoliation of germ cells from the epithelium. These findings are consistent with the known function of Map1a as an MT-associated protein by binding onto microtubules to promote MT stability. Its KD by RNAi rapidly disrupts the cytoskeletal organization of MTs across the Sertoli cell in a cell epithelium with an established functional TJ-barrier. Our findings are thus consistent with earlier reports on the functional significance of Map1a in maintaining MT function. For instance, mutations of the Map1 gene, nm2719, that disrupt the Map1a gene lead to cerebral Purkinje cell degeneration, due to degeneration of neuronal microtubules, causing tremors and ataxia as manifested by poor muscle control that causes difficulty with walking and balance [22]. Accumulation of Map1a was also found in differentiating embryonal carcinoma cells [97], and Map1a is also an emerging marker of bladder cancer [98]. Furthermore, considerably reduced expression of Map1a was detected in the hippocampus of patients with major depressive disorder during post-mortem examination by qPCR [99]. More importantly, similar dysregulation of Map1a expression was also detected in a rat model of chronic stress, and such down-regulation of Map1a was reversed by antidepressant treatment [99]. Such changes are likely the result of disruptive MT-dependent axonal nerve signaling transmission [99]. Collectively, these findings suggest that a crucial balance of the expression level of Map1a is necessary to maintain cellular homeostasis to avoid the development of cellular pathogenesis. This notion, in fact, is supported by the observation that a KD of Map1a in Sertoli cells perturbs not only MT organization across the cell cytoplasm but also actin organization as these linear MT and actin filaments no longer stretch across the entire cell but retract from cell peripheries. These changes are likely the results of mis-distribution of the actin regulatory proteins, such as Arp3, and Eps8. But possibly the MT regulatory proteins, such as EB1, and CAMSAP2, the corresponding MT plus-end tracking protein (+ TIP) and MT minus-end targeting protein (-TIP), and the various isoforms of α-tubulin that either promote MT stabilization (e.g., detyrosinated α-tubulin, acetylated α-tubulin) or MT dynamics (e.g., tyrosinated) [63, 100]. Furthermore, KD of Map1a also impedes the ability of Sertoli cells to polymerize MTs and actin filaments based on corresponding biochemical assays.

Using RNA sequencing and bioinformatics approaches, we have also identified a few signaling proteins that may be involved in modulating Map1a function in the testis as reported herein. Most notably is the activation of p-p38-MAPK during cadmium-induced Sertoli cell injury. However, the use of a selective inhibitor, doramapimod, specific to block p38-MAPK activation (i.e., phosphorylation) was capable of blocking the cadmium-mediated disruptive organization of the MT cytoskeleton. This finding is physiologically important since it illustrates that MT organization involving Map1a is supported by a defined signaling cascade based on p38-MAPK. Interestingly, this observation is in agreement with recent studies supporting the involvement of p38 MAPK in MT-dependent synaptic development of axons and motor protein dynein-mediated proteasome transport [101]; and p38 MAPK is implicated in Map4 mediated MT depolymerization in human vascular endothelium [102]. Studies based on findings noted in the datasets reported here have been deposited in the Public Domain, and studies are ongoing using these datasets to expand our current observations. It is likely that many unexpected outcomes will emerge regarding the biology of Maps in regulating spermatogenesis in the years to come.

In summary, we have shown that cadmium-induced testis and Sertoli cell injury based on the use of both in vivo and in vitro models are mediated through changes in the cytoskeletal organization of MT and actin. More important, cadmium exerts its disruptive effects through an activation (phosphorylation) of p38-MAPK, forming p-p38-MAPK, and the use of a specific inhibitor doramapimod effectively restores cadmium-mediated MT and actin cytoskeletal organization. These observations thus warrant future clinical investigations in men such as among those having industrial exposure of cadmium that hampered their reproductive function.

### Supplementary Information

**Supplementary Material 1.**

## Data Availability

All bulk RNA-Seq datasets reported in this study have been deposited in the NCBI as Sequence Read Archive data with signal recognition particle database SRP421648 at: https://www.ncbi.nlm.nih.gov/sra/?term=SRP421648. These include the 6 datasets with the following accession numbers: SRX19320913, SRX19320914, SRX19320915, SRX 19320916, SRX19320917, and SRX19320918, freely available for download.
