# Supplementary material for: Map-1a regulates Sertoli cell BTB dynamics through the cytoskeletal organization of microtubule and F-actin
Source: Reprod Biol Endocrinol. 2024 Apr 3;22:36. doi: 10.1186/s12958-024-01204-y (PMC10988971; doi:10.1186/s12958-024-01204-y)
Supplement: Supplementary file 1 — Supplementary Material 1. [file 12958_2024_1204_MOESM1_ESM.pdf]

Table S1. Antibodies used for different experiments reported in Figure S1

Antibody (RRID)	Host Species	Vendor	Catalog Number	IB Analysis (Working Dilution)
FAK (AB_732300)	Rabbit	Abcam	Ab40794	1:125
p-FAK-Y576 (AB_1310206)	Rabbit	Abcam	ab76120	1:500
p-FAK-Y407 (AB_2533708)	Rabbit	Invitrogen	44-650G	1:500
p-FAK-Y397 (AB_2533702)	Rabbit	Invitrogen	44-625G	1:500
Akt (AB_329827)	Rabbit	Cell Signaling Technology	9272	1:500
p-Akt1-T308 (AB_2255933)	Rabbit	Cell Signaling Technology	2965	1:500
p-Akt1-S473 (AB_2315049)	Rabbit	Cell Signaling Technology	4060	1:500
p-Akt2-S474 (AB_2630347)	Rabbit	Cell Signaling Technology	8599	1:500
rpS6 (AB_331355)	Rabbit	Cell Signaling Technology	2217	1:1000
p-rpS6-S235/S236 (AB_916156)	Rabbit	Cell Signaling Technology	4858	1:500
p-rpS6-S240/S244 (AB_10694233)	Rabbit	Cell Signaling Technology	5364	1:1000
mTOR (AB_330978)	Rabbit	Cell Signaling Technology	2972	1:500
p-mTOR-S2448 (AB_330970)	Rabbit	Cell Signaling Technology	2971	1:500
p-mTOR-S2441 (AB_2262884)	Rabbit	Cell Signaling Technology	2974	1:500
Prickle1 (AB_2718582)	Rabbit	Invitrogen	PA5-72728	1:500
Vangl2 (AB_2772841)	Rabbit	Abclonal	A7825	1:250
Fzd3 (AB_2757585)	Rabbit	Abclonal	A10063	1:250
Inversin (AB_2233902)	Rabbit	Proteintech	10585-1-AP	1:500
Dvl3 (AB_10694060)	Rabbit	Cell Signaling Technology	3218	1:500
ANKRD6 (AB_2879498)	Rabbit	Proteintech	24333-1-AP	1:500
Fjx1 (AB_2878406)	Rabbit	Proteintech	17417-1-AP	1:500
GAPDH (AB_2107436)	Mouse	Proteintech	60004-1-Ig	1:3000
β-actin (AB_630836)	Mouse	Santa Cruz Biotechnology	sc-4778	1:1000
Goat Anti-Mouse IgG H&L (Alexa Fluor® 680)	Mouse	Abcam	ab175775	1:3000
Goat Anti-Rabbit IgG H&L (IRDye® 800)	Rabbit	Abcam	ab216773	1:3000
Goat Anti-Rabbit IgG H&L (Alexa Fluor® 680)	Rabbit	Abcam	ab175773	1:3000
Goat Anti-Mouse IgG H&L (IRDye® 800)	Mouse	Abcam	ab216772	1:3000

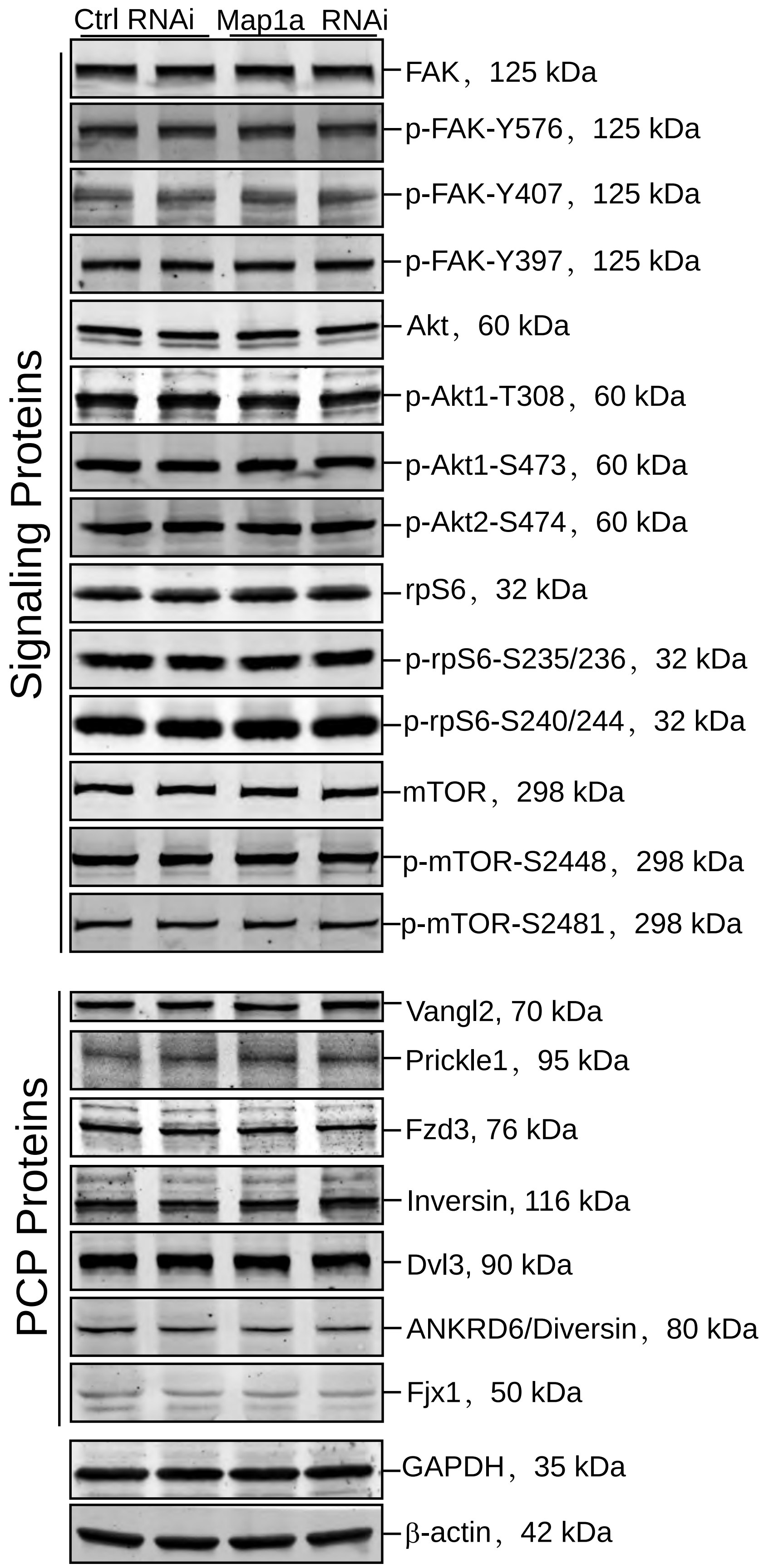

Figure S1. Knock-down (KD) of Map1a by RNAi does not affect the expression of virtually all the signaling and planar cell polarity (PCP) proteins. The signaling proteins selected for examination as reported here are earlier shown to be involved in the regulation of cytoskeletal dynamics of either actin or microtubule network, or both. These findings also support the notion that changes in the phenotypes (e.g., cytoskeletal organization of either actin or microtubule network) and/or functional status (e.g., tight junction permeability barrier function) of Sertoli cells were not the results of off-target effects of Map1a RNAi.

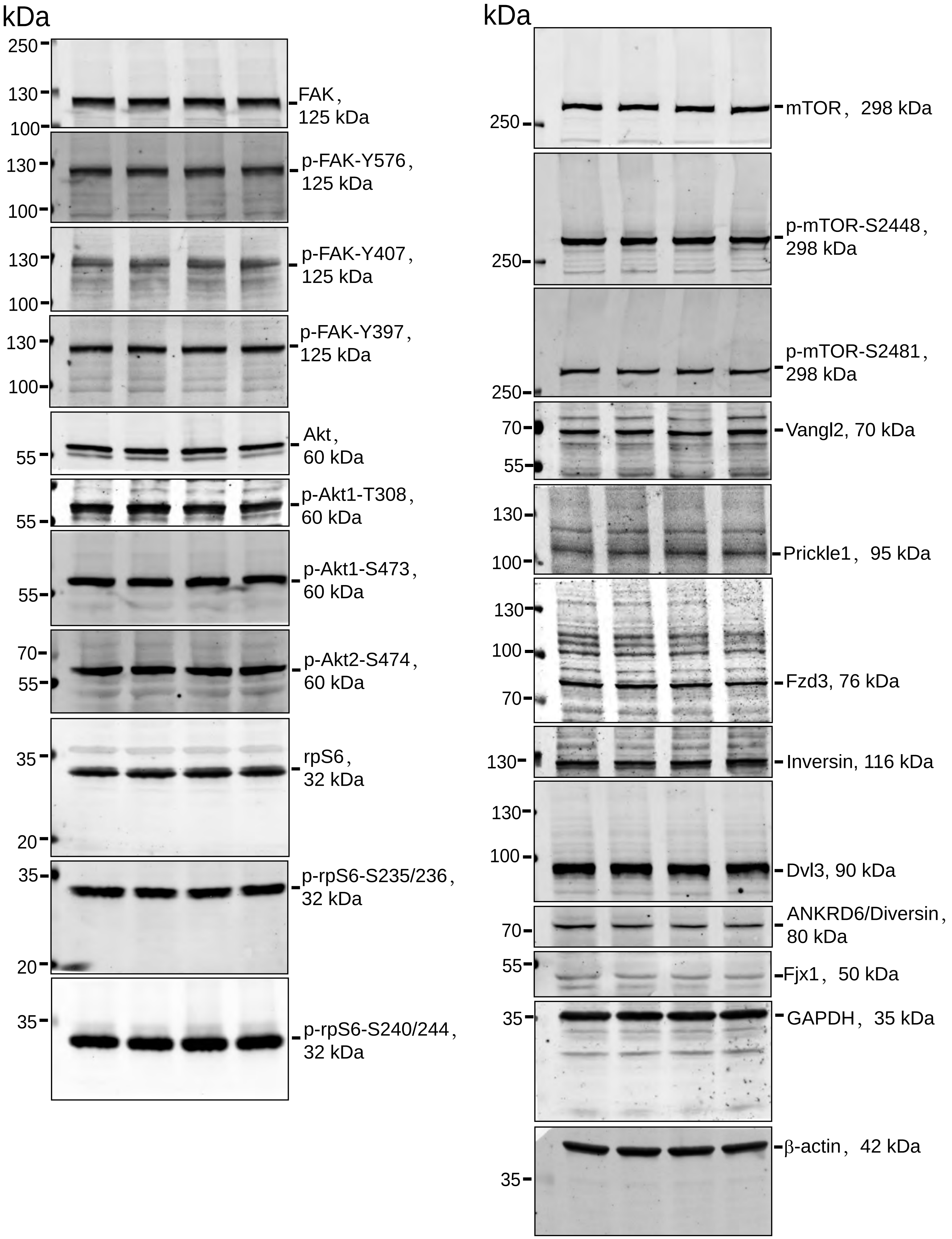

Figure S2. Immunoblots (IBs) shown here are the original blots that correspond to the cropped IBs of Figure S1.

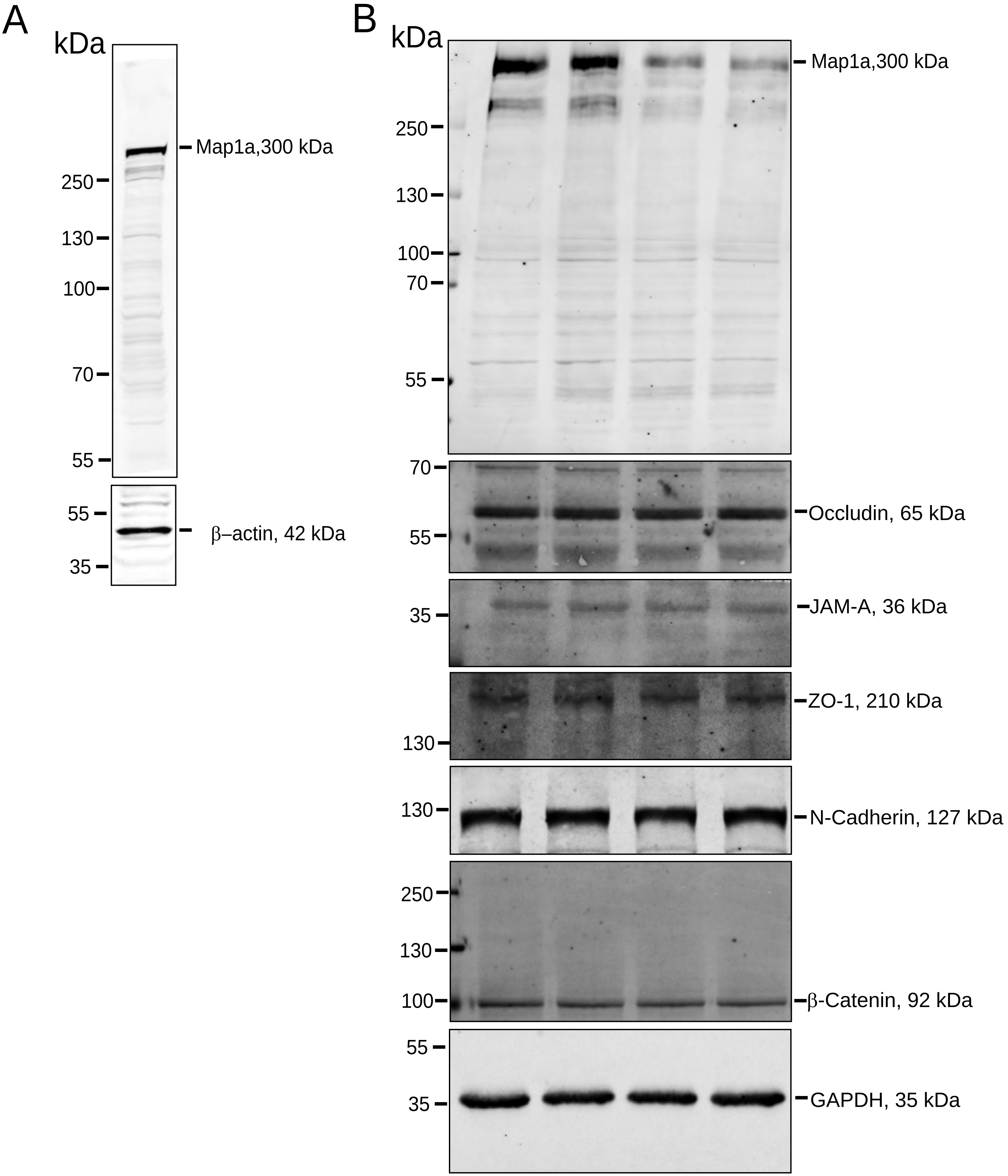

Figure S3. Immunoblots (IBs) shown in (A) and (B) are the original blots that correspond to the cropped IBs of Figure 1C and Figure 5C, respectively.

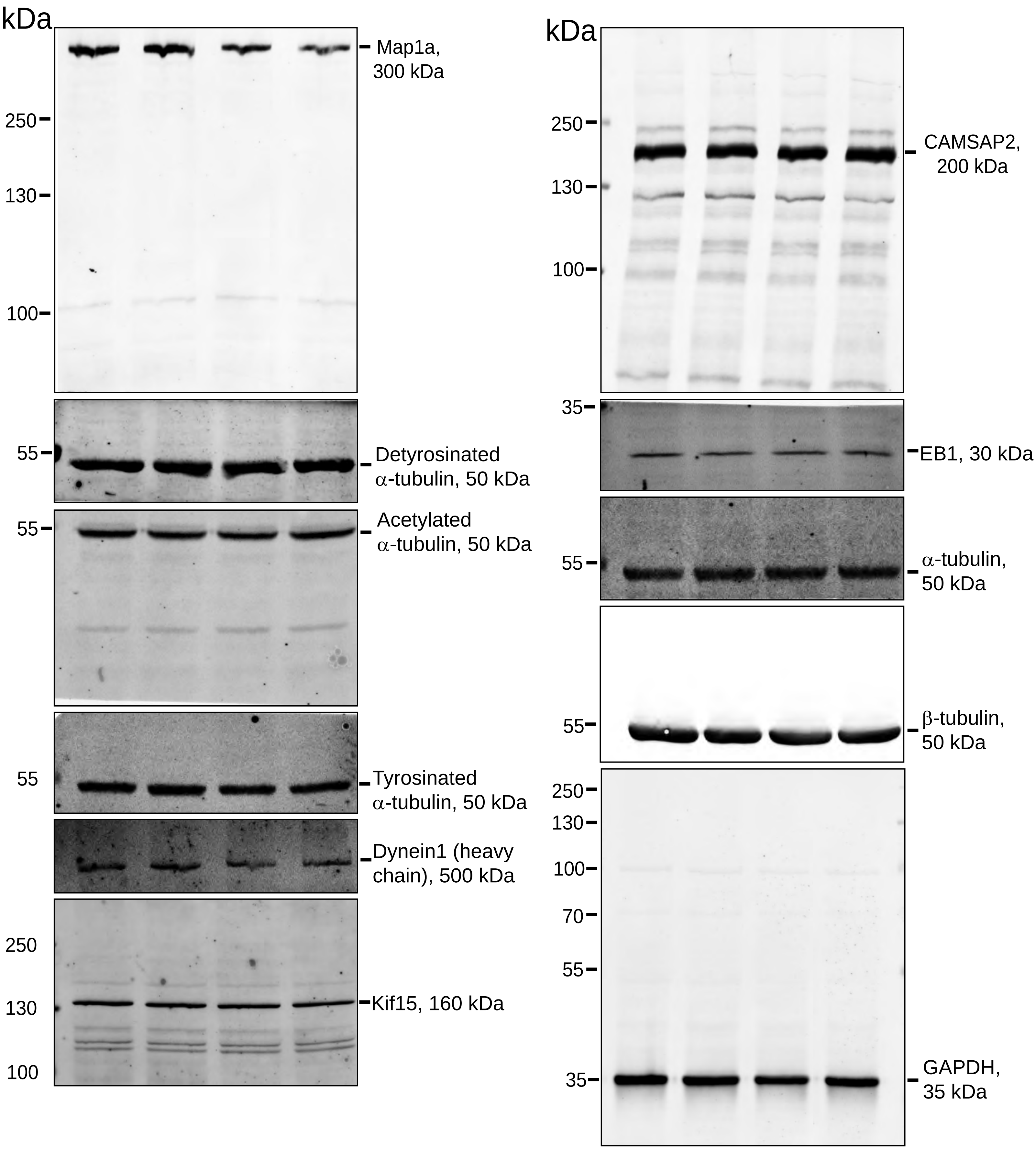

Figure S4. Immunoblots (IBs) shown here are the original blots that correspond to the cropped IBs of Figure 6B.

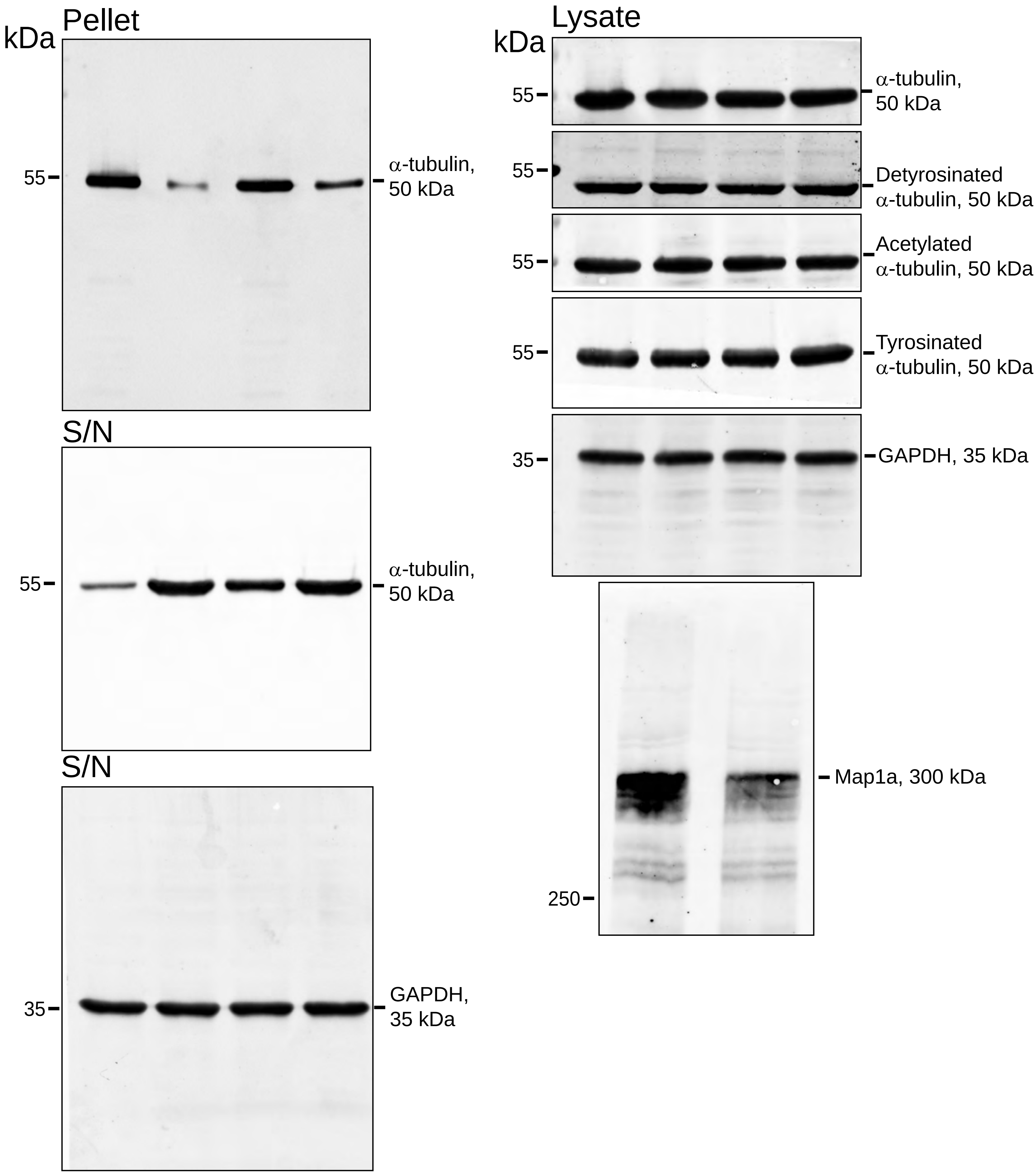

Figure S5. Immunoblots (IBs) shown here are the original blots that correspond to the cropped IBs of Figure 6C.

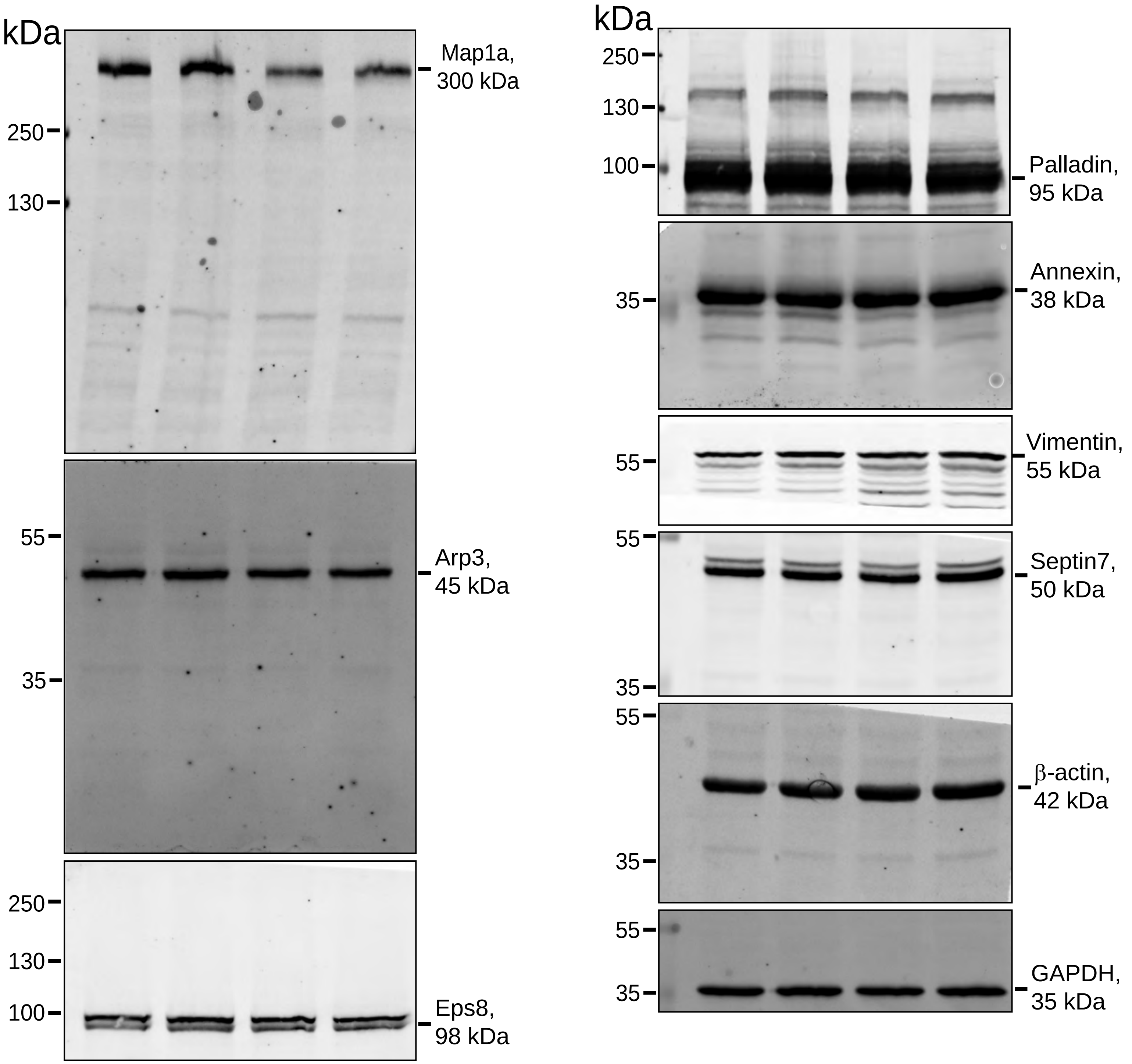

Figure S6. Immunoblots (IBs) shown here are the original blots that correspond to the cropped IBs of Figure 7B.

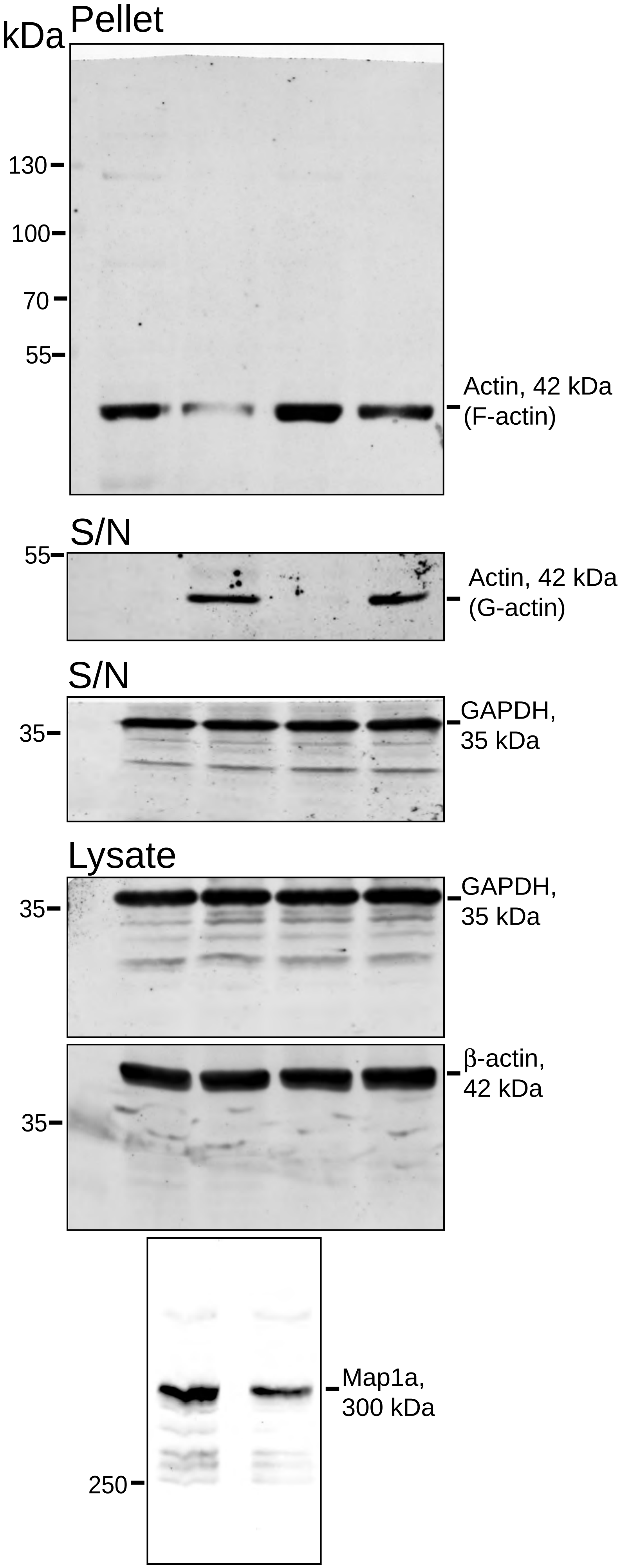

Figure S7. Immunoblots (IBs) shown here are the original blots that correspond to the cropped IBs of Figure 7C.

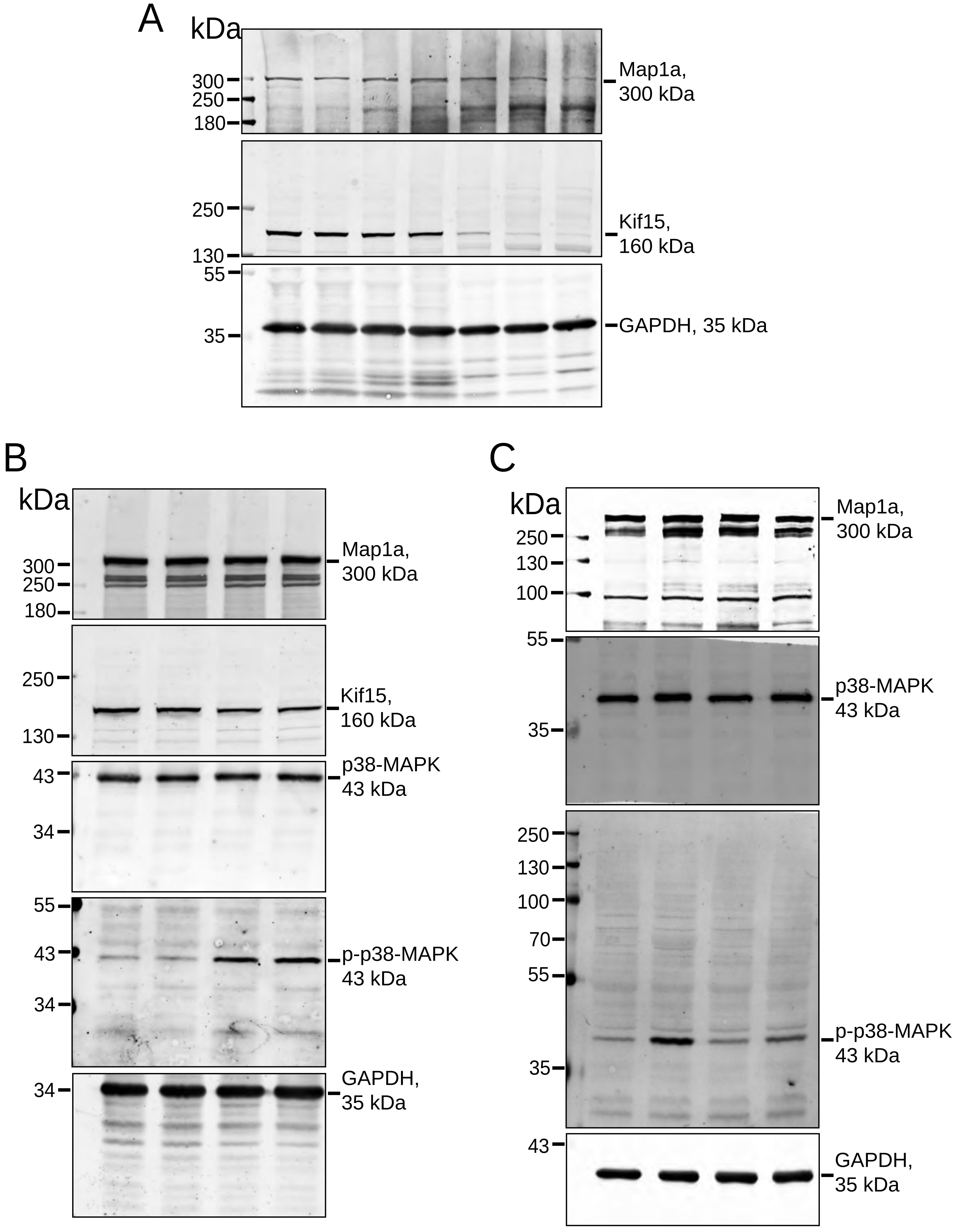

Figure S8. Immunoblots (IBs) shown in (A), (B), and (C) here are the original blots that correspond to the cropped IBs of Figure 8A, Figure 9D, and Figure 10B, respectively.
